# Elucidation of the Natural Function of Sophorolipids Produced by *Starmerella bombicola*

**DOI:** 10.3390/jof7110917

**Published:** 2021-10-28

**Authors:** Veerle De Clercq, Sophie L. K. W. Roelants, Martijn G. Castelein, Sofie L. De Maeseneire, Wim K. Soetaert

**Affiliations:** Centre for Industrial Biotechnology and Biocatalysis (InBio.be), Department of Biotechnology, Faculty of Bioscience Engineering, Ghent University, Coupure Links 653, 9000 Ghent, Belgium; veedcler.DeClercq@UGent.be (V.D.C.); Martijn.Castelein@UGent.be (M.G.C.); Sofie.DeMaeseneire@UGent.be (S.L.D.M.); Wim.Soetaert@UGent.be (W.K.S.)

**Keywords:** *Starmerella bombicola*, biosurfactants, sophorolipids, physiological function, natural role, antimicrobial, exclusive storage compound

## Abstract

The yeast *Starmerella bombicola* distinguishes itself from other yeasts by its potential of producing copious amounts of the secondary metabolites sophorolipids (SLs): these are glycolipid biosurfactants composed out of a(n) (acetylated) sophorose moiety and a lipid tail. Although SLs are the subject of numerous research papers and have been commercialized, e.g., in eco-friendly cleaning solutions, the natural function of SLs still remains elusive. This research article investigates several hypotheses for why *S. bombicola* invests that much energy in the production of SLs, and we conclude that the main natural function of SLs in *S. bombicola* is niche protection: (1) the extracellular storage of an energy-rich, yet metabolically less accessible carbon source that can be utilized by *S. bombicola* upon conditions of starvation with (2) antimicrobial properties. In this way, *S. bombicola* creates a dual advantage in competition with other microorganisms. Additionally, SLs can expedite growth on rapeseed oil, composed of triacylglycerols which are hydrophobic substrates present in the yeasts’ environment, for a non-SL producing strain (*Δcyp52M1*). It was also found that—at least under lab conditions—SLs do not provide protection against high osmotic pressure prevalent in sugar-rich environments such as honey or nectar present in the natural habitat of *S. bombicola*.

## 1. Introduction

**Figure 1:**
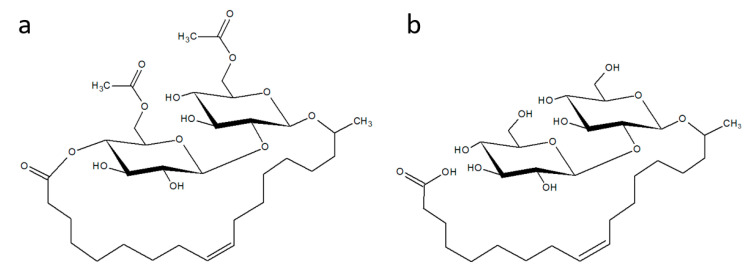
The structure of a lactonic (**a**) and an acidic (**b**) sophorolipid (SL), produced by (amongst others) *Starmerella bombicola*. Both glucose molecules can be acetylated on position C6 as shown in (**a**); these acetyl groups can be completely absent as shown in (**b**), but also mono-acetylated SLs are produced.

The name ‘*Starmerella bombicola*’ strongly associates with ‘biosurfactants’ (bio-based surface-active agents), as this species is the best known producer of sophorolipids, which are secondary metabolites: more than 75% of the research publications on this yeast focus on sophorolipids [1,2]. Sophorolipids (SLs) are amphiphilic molecules with a hydrophilic sophorose head, consisting of two β-1,2 linked glucose molecules, and a hydrophobic fatty acid tail. The SLs produced by *S. bombicola* are typically a mixture of several slightly different congeners providing each of them with specific properties [3,4,5,6,7,8]. For example, SL molecules with a free carboxylic group are called acidic SLs and have good solubility in water. This carboxyl group can be esterified with the hydroxyl group on the C4” of sophorose forming a macrocyclic ring giving rise to lactonic SLs, which are characterized by low water solubility. Examples of other diversifications are: the length and saturation degree of the fatty acid chain (for *S. bombicola*, mainly C16:1 or C18:1), the position of attachment of the sophorose moiety (through a glycosidic bond) to the fatty acid at the ω (terminal) or ω-1 (sub-terminal) position, and the acetylation degree of sophorose (major structures are shown in Figure 1) [3,9,10].

The ‘soap-like’ structure of SLs provides them with wetting, emulsification, foaming, and dispersing properties combined with a sustainable and environmental-friendly character. These properties make them attractive for the detergent industry, medical world, personal care sector, mining processes, food industry, cosmetics, crop protection, pharmaceuticals, and bio-remediation [11,12,13,14,15,16,17]. This broad industrial relevance attracts the attention of many researchers, resulting in ample and diverse application investigations [2]. On the other hand, several laboratory tools were developed over the past decade, gradually turning *S. bombicola* into an engineerable organism and, as such, expanding the portfolio of molecular structures and concomitant functions and applications [16,18,19,20,21]. Although the research on *S. bombicola* and its produced molecules has given rise to an exponential growth of research papers on the subject, the natural function of sophorolipids in *Starmerella bombicola* still remains obscure [1].

The aim of this study is to resolve this fascinating microbial mystery in a rational manner; the answer to the question ‘why does *S. bombicola* produce SLs?’ should be related to improved fitness, yielding in an immediate or delayed growth benefit. The fact that SLs are secondary metabolites indicates they do not fulfill (an) essential function(s) in cell growth and/or maintenance, but their presence favors the producing organism in specific conditions. A first aspect that needs to be considered is the natural environment of *S. bombicola* and its associated characteristics. *S. bombicola* was isolated from nectar samples of wild flowers (in 1954) and bumblebee honey (1964–1967) by Spencer et al. [22]. They identified this new yeast species as *Torulopsis bombicola* due to its close association with bumblebees. Later on, the yeast was reclassified as *Candida bombicola* and finally renamed *Starmerella bombicola* in 2012 [23,24]. In the years following the discovery, additional *S. bombicola* strains were isolated all over the world, primarily originating from samples derived from flowers and (bumble)bees (and exceptionally also from some other flower visiting insects) [2]. The associated physical and biological factors characterizing these habitats and affecting the microorganisms colonizing it are mainly: high osmotic pressure (low water activity) due to the abundance of sugars in honey (and in lesser concentrations also in nectar) (see Table 1), floral microhabitats with dynamic microbial communities due to high dispersal and foraging by (pollinating) visitors, and the availability of a wide variety of substrates including hydrophobic substrates—such as (mono)esters of long chain fatty acids in beeswax and triacylglycerols in plant oils of flower seeds [25,26,27,28,29].

Several plausible hypotheses on the natural role of SLs in *Starmerella bombicola* have been postulated in the literature and can be summarized as follows: (1) SL production constitutes an overflow metabolism [30]; (2) SLs exert antimicrobial activity, thereby inhibiting the growth of competing microorganisms [15,31,32,33,34,35,36,37]; (3) SL production is a protection mechanism against high osmotic pressure [38]; (4) SLs improve the uptake of hydrophobic substrates [39,40]; and (5) SLs serve as an extracellular storage compound of carbon and energy [38].

The first hypothesis (1) that SL production constitutes an overflow metabolism in order to regulate intracellular energy levels was proposed by Davila et al. [30]. This theory can be easily rejected based on convenient knowledge: overflow metabolism relates to a deficit in the cofactor NAD^+^, which cannot be regenerated through the biosynthesis of SLs [41]; an elaborated argumentation can be found in Dierickx et al. [42]. The latter three hypotheses (3–5) are rather speculative without substantiated data and are therefore investigated in this research article, together with the hypothesis on the antimicrobial activity (2) for which some preliminary data exist. This study is the first one to answer the question ‘Why does *S. bombicola* produce SLs?’ in a more thorough way. According to the findings, SL production is not accompanied by elevated protection against high osmotic pressure arising from high sugar concentrations. It was found however that *S. bombicola* can catabolize its previously synthesized SLs in times of starvation, and the antimicrobial activity was confirmed. We conclude that the main natural function of SLs for *S. bombicola* can be defined as niche protection through a dual advantage: the build-up of an exclusive and extracellular storage compound, characterized by antimicrobial properties. As an additional advantage, *S. bombicola* could possibly benefit from SLs in the presence of hydrophobic substrates (e.g., triacylglycerols), as improved growth rates were noticed on rapeseed oil for the SL deficient *Δcyp52M1* strain by adding SLs (but an adverse effect was found for the alkane hexadecane).

## 2. Materials and Methods

### 2.1. In Vitro Antimicrobial Assay

For the antimicrobial activity of SLs, the following wild-type laboratory strains were chosen: Gram-positive bacteria (*Bacillus subtilis* LMG 2099, *Staphylococcus aureus* LMG 8224, *Fructobacillus fructosus* LMG 30235), Gram-negative bacteria (*Escherichia coli* MG 1655, *Pseudomonas aeruginosa* LMG 24986, *Hafnia alvei* LMG 28933), and yeast species (*Candida albicans* SC 5314, *Zygosaccharomyces rouxii* MUCL 30008, *Starmerella bombicola* ATCC 22214). Precultures of *E. coli, P.* *aeruginosa*, and *B. subtilis* were grown on Lysogeny broth (10 g·L^−1^ tryptone, 5 g·L^−1^ yeast extract and 5 g·L^−1^ NaCl); *H. alvei* was cultivated on nutrient broth (Oxoid); *S. aureus* was cultivated on brain heart infusion broth (Biokar diagnostics); *F. fructosus* was cultivated on MRS broth (Thermo Fisher Scientific); *C. albicans* and *S. bombicola* were cultivated on 3C medium (100 g·L^−1^ glucose, 10 g·L^−1^ yeast extract, and 1 g·L^−1^ urea), and *Z. rouxii* was cultivated on YPD medium (5 g·L^−1^ yeast extract, 10 g·L^−1^ peptone and 20 g·L^−1^ glucose). All bacterial precultures, except for *F. fructosus* and *H. alvei*, were cultivated at 37 °C and 200 rpm for 24 to 36 h. *H. alvei, F. fructosus,* and the yeast strains were grown at 30 °C and 200 rpm for 48 to 72 h.

For the antimicrobial assay, Mueller Hinton broth (MHB, Biokar diagnostics) was used at a pH of 4 for yeasts and a pH of 7 for bacteria using a citrate-phosphate buffer (McIlvaine buffer, 0.1 to 0.2 M depending on the pH setpoint). Precultures were diluted in MHB to a turbidity of the 0.5 McFarland standard, which equals approximately 1–2 × 10^8^ CFU·mL^−1^, and were subsequently inoculated at 1.3% in 96-well microtiter plates (MTP) (Greiner Bio-One) with a total volume of 150 µL MHB with varying SL concentrations ranging from 0.5 g·L^−1^ up to 20 or 30 g·L^−1^. These MTPs were incubated for 24 h on a MTP shaker (700 rpm) at 30 °C (yeasts) or 37 °C (bacteria). Due to growth difficulties on MHB for *Z. rouxii* and *F. fructosus*, aberrant media/conditions were used; these strains were cultivated on a YPD or MRS medium, respectively, at 30 °C for 48 h.

Three different C18:1/C18:0 sophorolipid (SL) compounds (purity ~99%) were evaluated for their antimicrobial activity (prepared as described below in 2.3): non-acetylated acidic SLs (uniformity 99.7%), di-acetylated lactonic SL (uniformity 95.0%), and a wild-type SL mixture. SLs were prepared as 200 g·L^−1^ (acidic and lactonic SLs) or 300 g·L^−1^ (wild-type SL mixture) stock solutions in dimethyl sulfoxide (DMSO) to circumvent solubility issues. Therefore, a preliminary DMSO serial dilution test (up to 10% *v*/*v*) was used to evaluate microbial viability upon the DMSO addition. The maximum tested SL concentration per microbial strain depended on the highest DMSO concentration that did not affect growth of that strain, with a maximum SL concentration of 20 g·L^−1^ (acidic and lactonic SLs) or 30 g·L^−1^ (wild-type SL mixture), corresponding to a DMSO concentration of 10%. Per strain, the determined maximum DMSO concentration that should not affect growth was included in parallel in the assay as a control.

Minimum inhibitory concentration (MIC) and minimum lethal concentration (MLC) values were determined in duplicate (*n* = 2) using a 96-well plate serial dilution method based on Clinical and Laboratory Standards Institute (CLSI) guidelines [43,44,45]. A two-fold serial dilution of SLs ranging from ~0.5 g·L^−1^ up to 20 or 30 g·L^−1^ (or lower in the case of DMSO sensitive organisms) was made by mixing the SL stock solution (20 or 30 g·L^−1^) with the testing medium (MHB, MRS, or YPD) with a total volume of 148 µL and was subsequently inoculated (total volume of 150 µL). During cultivation (24 or 48 h), growth was monitored by optical density measurements at 600 nm every 7 h (FLUOstar OPTIMA FL, BMG Labtech). As (lactonic) SLs interfere with OD_600_ measurements, growth was also evaluated using a resazurin assay [46]. This fluorometric/colorimetric assay is based on the reduction of the blue dye resazurin (absorbance at 600 nm) with a formation of the red fluorescent dye resofurin (ex/em of 530–560 nm/590 nm) by metabolic active cells. In practice, 30 µL of a 0.2 g·L^−1^ filter sterilized resazurin solution (Sigma-Aldrich) was added to the cell cultures in the MTP after 24 or 48 h of cultivation and incubated for 2 extra hours. Microbial growth was verified through fluorescence measurements of resorufin at 560 nm/590 nm (FLUOstar OPTIMA FL, BMG Labtech). MIC values were assigned as the lowest concentration of SLs inhibiting growth of the microorganisms (evaluated through OD_600_ and fluorescence measurements), while MLC values were determined as the lowest SL concentration at which no microbial growth was observed anymore after inoculation into the fresh medium (MHB, MRS, or YPD).

### 2.2. Strains, Media, and Culture Conditions

In this study, the wild-type strain *Starmerella bombicola* ATCC 22214, a *S. bombicola* Δ*cyp52M1* strain deficient in the first step of SL biosynthesis [47], and a *S. bombicola* Δ*sble* strain [48,49] were used. Yeast cells were maintained on the YPD medium (10 g·L^−1^ yeast extract, 20 g·L^−1^ peptone, and 20 g·L^−1^ glucose) or 3C medium (100 g·L^−1^ glucose, 10 g·L^−1^ yeast extract, and 1 g·L^−1^ urea) with the addition of 20 g·L^−1^ agar (Biokar diagnostics) when required.

Shake flask experiments for the production of sophorolipids (SLs) (30 °C and 200 rpm) were performed with the wild-type *Starmerella bombicola* ATCC 22214 strain in the production medium described by Lang et al. (see ‘production medium’ in Table 2) to which 3.75% rapeseed oil (from a local supermarket) (or 3.75% oleic acid when specified, Sigma-Aldrich) was added after 48 h [50]. The cultivation was stopped after 2 weeks (after confirmation that all of the hydrophobic substrate was consumed). The SLs were purified from these cultures as described below.

The growth experiments to investigate the effect of high osmotic pressure were executed in 96-well microtiter plates (MTP) (Greiner Bio-One) with 200 µL of the SD CSM medium (Synthetic Defined medium with Complete Supplement Mixture) supplemented with varying glucose or fructose concentrations (ranging from 20 to 600 g·L^−1^). The composition of the SD CSM medium with 20 g·L^−1^ glucose can be found in Table 2. All components were filter sterilized separately. For preculture preparation, six single colonies of each strain (*n* = 6) were transferred from YPD plates into 200 µL of the SD CSM medium supplied with 100 g·L^−1^ glucose or fructose, incubated at 30 °C for 2 days on a MTP shaker (800 rpm) and subsequently used to inoculate the final MTP at 1% (total well volume 200 µL). The final plate was incubated in an Infinite 200 PRO multimode reader (Tecan) for 60 h at 30 °C and orbital shaking with an amplitude of 2 mm.

To examine growth on hydrophobic substrates, precultures were prepared from three single colonies on YPD plates in test tubes containing 5 mL of the SD CSM medium supplemented with only 2 g·L^−1^ of glucose (instead of 20 g·L^−1^) to avoid carry over of glucose to the final experiment. A synthetic defined medium was used to minimize growth on other medium compounds, which was confirmed by a lack of growth in the negative controls (SD CSM medium without C-source) for at least 7 days. After 3 days of growth, 1 mL of the preculture was centrifuged; the cell pellets were washed (to avoid potential carry over of the remaining glucose or produced SLs, confirmed with TLC) and subsequently resolved with a sterile physiological solution. The volumes of the physiological solution were chosen such to obtain equal final cell concentrations (based on OD_600_ values). Finally, the cells were inoculated at 3% into test tubes containing 5 mL of the SD CSM medium (Table 2) supplemented with 20 g·L^−1^ of three different hydrophobic carbon sources as the sole C-source (in duplicate or triplicate): beeswax (Weyn’s honing; *n* = 2), hexadecane (Sigma-Aldrich, *n* = 3), or rapeseed oil (from a local supermarket, *n* = 3). Positive and negative controls consisted of the SD CSM medium with 20 g·L^−1^ glucose and the SD CSM medium without any C-source (no glucose), respectively. All components were autoclaved separately. A sterile wild-type sophorolipid mixture (prepared as described below) was added to one of two replicas of the same colony in a concentration of 1 g·L^−1^. The cultures were incubated for 14 days at 30 °C and 200 rpm.

An adapted medium was used when growth on SLs (di-acetylated lactonic) as the sole C-source was envisaged. This growth medium will be called the SL medium and can be found in Table 2. The SLs (produced as described below) were added to the medium components, and the mixtures were subsequently filter sterilized (Corning). Due to growth difficulties and to prepare the cells to metabolize SLs, an alternative method was used to start up precultures for the culture on the SL medium: 5 mL of an old culture on the production medium (cultivated for >20 days) were spun down, and the harvested cells were dissolved in 5 mL of the SL medium. After incubation at 30 °C and 200 rpm for at least 24 h (until microscopic observations showed that cells were clearly budding), this culture was used as a second inoculum to prepare a second preculture on the SL medium (5 mL), which was in turn used as an inoculum for a shake flask experiment on the SL medium (200 mL, *n* = 3). For the experiment investigating SL degradation during prolonged cultivation on the production medium, three 1 L shake flasks with 200 mL of the medium were inoculated (2%) with overnight grown precultures, prepared from three single colonies (*n* = 3) on a 3C plate in 5 mL of the production medium.

### 2.3. Preparation of (Partly) Purified Sophorolipids

Sophorolipids (SLs) were produced by the wild-type strain *Starmerella bombicola* ATCC 22214 in the production medium (with the addition of rapeseed oil, see above). Analytics were performed as described in Section 2.7.

Di-acetylated lactonic SLs (C18:1) were prepared in a very pure (purity ~99%) white powder (uniformity 95.0%) via crystallization, and non-acetylated acidic SLs (C18:1) (uniformity 99.7%) were derived from lactonic SLs through alkaline hydrolysis and purified as described by Roelants et al. [49].

To produce the wild-type SL mixture, used for the hydrophobic substrate experiment and the in vitro antimicrobial assay, oleic acid was added as the hydrophobic substrate. The wild-type SL mixture was partly purified through melting as described by Roelants et al. [49]: the production broth was heated to 70 °C in a separation funnel, and the melted SLs collected at the bottom. SLs were tapped off and thoroughly washed with dH_2_O. This process was repeated with the washed SL mixture. The final composition consisted of 88% C18:1 di-acetylated lactonic SLs, 2% C18:0 di-acetylated lactonic SLs, 7% C18:1 mono-acetylated lactonic SLs, and 3% C18:1 di-acetylated acidic SL (based on the peak area from ELSD); there were no leftover traces of the oleic acid substrate.

### 2.4. Sampling and Determination of Growth Parameters

The effect of high osmotic pressure was investigated with continuous OD_600_ measurements in an Infinite 200 PRO multimode reader (Tecan) and was based on six replicates (*n* = 6). The growth parameters were determined in R (4.0.2) by using smoothing splines (for maximal growth rate µ_max_) and the Gompertz function (for the determination of the lag phase and ΔOD) from the ‘growthrates’ package (https://cran.r-project.org/web/packages/growthrates/vignettes/Introduction.html, accessed on 28 December 2019). As some of the sub-datasets were not normally distributed, the Mann–Whitney U test was used for statistical analysis.

For the growth experiment on hydrophobic substrates, the test tubes (duplicates for beeswax and triplicates for rapeseed oil and hexadecane) were sampled at regular time points (see Figure S5 for the timings): 75 µL of the broth were mixed with 75 µL of the physiological solution, and the OD_600_ of the solution was determined using a microplate reader (FLUOstar OPTIMA FL, BMG Labtech). The measured OD_600_ values were first corrected for the average optical density that was caused by the addition of sophorolipids to each substrate (so the average of the differences in OD values at t = 0 for each replica/colony for that substrate). Three growth parameters were estimated from the obtained log transformed growth curves: an approximately estimated growth rate µ, the estimated duration of the lag phase, and the ΔOD (which equals the grown number of cells). Due to the discontinuous sampling/measuring, the data were not fitted for the ‘growthrates’ package (see above). Therefore, smoothing splines were used in R (4.0.2) to estimate µ (the maximum first derivative to the smoothed spline) and the duration of the lag phase (the intersection of that estimated maximal slope line with the horizontal y = x_0_). The parameters ‘estimated µ’ and ‘lag’ of the growth curves that did not exceed the maximum corrected OD value of the negative controls (which are non-inoculated SD CSM media with beeswax, hexadecane, or rapeseed oil; and inoculated SD CSM media without the C-source, all with and without the addition of SLs) were set at µ = 0 and lag = Inf, respectively. For the sub-dataset with rapeseed oil as the substrate (*n* = 3), a paired t-test was used for the statistical analysis.

In the SL degradation experiments, three shake flask cultures (*n* = 3) were sampled regularly for cell concentration (CFU), glucose concentration, and pH determination; a supernatant collection was performed for the ammonium concentration and SL composition (see below). Glucose concentrations were determined using the 2700 select biochemistry analyzer (YSI Inc.); samples were diluted below 7.5 g·L^−1^ with dH_2_O. The amount of free ammonium (NH_4_^+^-ions) in the cell free culture medium was determined using the Ammonia Rapid Megazyme Kit. A standard solution was prepared using NH_4_Cl. The viability of yeast cells in prolonged cultivation experiments was assessed by determining colony forming units (CFU) on 3C agar plates (incubation at 30 °C for three days).

### 2.5. Assays with Extracellular Secretomes

Unconcentrated secretomes, i.e., cell free culture media containing all the extracellular proteins, were prepared in triplicate (*n* = 3) from *S. bombicola* cultures cultivated on the production medium for 21 days (see strains, media, and culture conditions). Centrifugation (15 min, 4000× *g*, 4 °C) of the culture broth was followed by an additional centrifugation step to remove all insolubles. The resulting cleared supernatant (200 mL) was divided into three volumes: the first volume was supplemented with di-acetylated lactonic SLs (20 g·L^−1^), the second volume with non-acetylated acidic SLs (20 g·L^−1^), and the last one with an equal amount of sterile dH_2_0 to control for the background of remaining SLs and/or other compounds in the protein solutions. The resulting solutions were filter sterilized using a 0.22 µm filter (Corning) and incubated (30 °C) in sterile shake flasks on a rotary shaker (200 rpm). Two flasks with the fresh production medium without glucose and set at the pH of the collected secretomes (pH = 5.5) were supplemented with both forms of SLs, respectively, and served as controls. The flasks were incubated for one month and sampled regularly for SL analysis, glucose, and pH determination.

For concentrated secretomes, three tablets of the cOmplete protease inhibitor cocktail (Roche) were added to 200 mL of the cleared supernatant (see above, *n* = 3), and the secretomes were subsequently filtered using a 0.22 µm filter. Secretomes were subsequently concentrated using a stirring ultra-filtration cell (Model 8200 Milipore) containing a 10 kDa cut off G membrane (Sartorius Stedim). After concentrating to 30 mL, one volume of wash buffer (20 mM Tris, pH 7) was added. The secretome was subsequently concentrated again to 30 mL. The protein concentration was determined using a BCA Protein Assay Kit (Pierce). In case the concentration was lower than 1 mg·mL^−1^, the secretomes were further concentrated using a Vivaspin 15R (Sartorius stedim). The obtained concentrated secretomes were divided into 2 mL Eppendorf tubes (1 mL at 1 mg·mL^−1^ protein) and supplemented with 0.5 mM MgCl_2_ and 2 mM of di-acetylated lactonic SLs (1.4 g·L^−1^) or non-acetylated acidic SLs (1.2 g·L^−1^). Two controls were included: a first control consisted of 2 mM of SLs in the wash buffer to check for a potential spontaneous breakdown of SLs. A second one consisted of the concentrated secretome preparations supplemented with dH20 (instead of SLs) to correct for the background of remaining SLs and/or other compounds in the protein solutions. All assays were incubated for 14 days (30 °C, on a rotary wheel at 30 rpm).

### 2.6. Assays with Intracellular Cell Lysates

Cell pellets of *S. bombicola* cultures (*n* = 3), cultivated on the production medium for 21 days, were collected by centrifugation (10 min, 4000× *g*, 4°C). The pellets were dissolved in a lysis buffer (20 mM Tris; pH 7; 0.5 mM MgCl_2_; 5% glycerol; and 1 tablet of cOmplete protease inhibitor cocktail (Roche) per 50 mL buffer) and added into a 2 mL tube of lysing matrix C (MP biomedicals) containing 1 mm silica spheres and subjected to 2 cycles of 6 m·s^−1^ in a FastPrep Celldisrupter (MP biomedicals). The crude lysates were subjected to centrifugation (10 min, 4000× *g*, 4 °C). The resulting supernatants (lysate preparations) were transferred into 2 mL Eppendorf tubes (1 mL at 1 mg·mL^−1^ protein), supplemented with 2 mM of SLs and incubated in triplicate (*n* = 3) at 30 °C on a rotary wheel at 30 rpm for 6 days for di-acetylated lactonic and 9 days for non-acetylated acidic SLs, respectively. For each assay, two controls were included as already described for the secretomes, summarized as follows: 2 mM of SLs in lysis buffer and dH_2_0 (instead of SLs) added to the lysate preparation.

### 2.7. Product Identification and Quantification

Analytical samples (except the samples of the concentrated assays, see below) were prepared by mixing 2 volumes of ethanol with 1 volume of the culture broth after which the mixture was shaken vigorously for 5 min. The mixture was centrifuged (10,000 rpm for 5 min), and the clear supernatant consisting of a EtOH/H_2_0/SL mixture was analyzed after filtration with a 13 mm PTFE syringe filter with a pore size of 0.2 μm (Novolab). For the samples of the assays (with concentrated secretomes and cell lysates), hydroxylated lauric acid (50 nmol) was added as an internal standard before extraction: 440 µL ethyl acetate and 11 µL acetic acid were added to 1 mL of the assay volume and shaken vigorously for 5 min. After centrifugation (10,000 rpm for 5 min), the upper solvent layer was removed and translocated into a fresh Eppendorf tube, containing 700 µL of ethanol and filtered before analysis.

HPLC-ELSD (Varian Prostar HPLC system) analyses were performed as described by Saerens et al. [51]. The same column and LC conditions were used for performing HPLC-MS analyses, using an Intertek ASG (Manchester, UK) with a Micromass Quattro Ultima LIMS1107 (Waters). The detection range was set at *m*/*z* 100 to 1000, and the negative ion mode was applied.

A second HPLC-MS method was used for sophorose detection. The used column was a Hypercarb PGC 100 × 4.6 mm column from Thermo. Three eluents were used: 4% methanol in ultrapure water (A), 100% acetonitrile (B), and 15% formic acid in ultrapure water (C) on a flow rate of 0.6 mL·min^−1^ and run time of 40 min. The analysis started at 100% of eluent A for 4 min, after which a linear decrease of A to 78% and respective increase of B to 22% were executed in 5 min. This was followed by a further decrease of A to 41% and respective increase of C to 37% in the next 8 min after which eluent A decreased further to 3% in the next 10 min, whereas C increased to 75% and B was kept at 22%. After this, A and B were increased again to 50% in 2 min, and C dropped back to 0%. The next minute served to raise A back to 100%, which was kept that way for the last 10 min of the run. A sophorose standard of 250 mM was used.

UPLC-ELSD (Waters Acquity H-class system) analyses were performed as described by Van Renterghem et al. [16]. For UPLC-HRMS (Thermo ScientificTM ExactiveTM Plus Orbitrap Mass Spectrometer), the same LC method was used, and high-resolution scanning happened in the negative ionization mode (ESI) for a mass range of 215 to 1800 *m*/*z*.

For a quick follow up of SL production and substrate use, TLC was performed on the culture broth samples (2 to 4 µL) as described by Van Renterghem et al. [16].

## 3. Results

As already mentioned in the introduction, five hypotheses on the natural role of sophorolipids (SLs) for *S. bombicola* have been postulated. Four of those statements were investigated in this paper: (1) the antimicrobial activity of SLs, (2) the protection against high osmotic pressure, (3) an improved uptake of hydrophobic substrates, and (4) an extracellular storage compound.

### 3.1. Antimicrobial Activity

SLs are claimed to possess antimicrobial activity and as such favor the producing organism against competing microorganisms occupying the same ecological niche. Antimicrobial activity was first reported for lactonic SLs that inhibited growth of specific alkane utilizing yeasts (only when grown on C10 to C18 alkanes) [31]. Succeeding studies showed that SLs also inhibit bacteria (e.g., *B. subtilis* and *P. aeruginosa*) and fungi (e.g., *U. maydis* or foodborne *Aspergillus* strains) [15,32,33,34,35,36,37]. Here, the antimicrobial properties of SLs were investigated against some standard microbial strains used for antimicrobial studies and representing different microbial groups, and three ecologically highly relevant strains.

**Figure 2:**
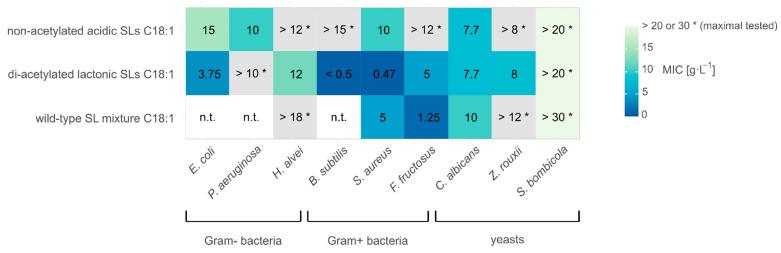
A heatmap of the minimum inhibitory concentrations (MIC) in g·L^−1^ found for the wild-type sophorolipid mixture (C18:1) and two of its (main) components: di-acetylated lactonic and non-acetylated acidic SLs. For the combinations marked with a * no MIC value could be determined within the tested concentration range (maximum tested value is depicted on the heatmap), and combinations that were not tested are designated as n.t. MIC values were determined in duplicate (*n* = 2) and were equal for each replicate.

An in vitro antimicrobial assay was thus performed to determine the minimum inhibitory concentrations (MIC) of a *S. bombicola* wild-type SL mixture (acidic and lactonic SLs) and two purified components: di-acetylated lactonic SLs and non-acetylated acidic SLs. Especially purified lactonic SLs are interesting to test, as they are the main products that are found extracellularly, where they should exert their antimicrobial activity as a natural function. The antimicrobial activity was tested against species belonging to genera that are associated with flowers and (bumble)bees: Gram+ bacteria (*B. subtilis, S. aureus, F. fructosus*), Gram- bacteria (*E. coli, P. aeruginosa, H. alvei*), and yeasts (*C. albicans, Z. rouxii*) (see Figure 2) [52,53,54,55]. Three strains in particular are natural competitors in the ecological niche of *S. bombicola* as they were all isolated from the gut of bumblebees (and from honey and/or floral nectar): *Fructobacillus fructosus, Hafnia alvei*, and *Zygosaccharomyces rouxii* [56,57,58]. Due to solubilization difficulties in water, SLs were dissolved in DMSO. To check whether the inhibitory effect could be attributed to the SLs and not to DMSO, a preliminary test was performed for each strain to determine the maximal DMSO concentration that did not affect growth. These concentrations of DMSO were included in the assays as controls. Since the pure DMSO concentrations corresponding to the maximum tested SL concentrations did not inhibit growth (data not shown), the observed inhibitory effects can be attributed to the SLs.

In general, the lactonic SLs exerted the highest antimicrobial activity, while acidic SLs or the wild-type SL mixture showed the strongest inhibition for *P. aeruginosa* and *F. fructosus*, respectively. MLC values (minimum lethal concentration) could only be determined for *S. aureus, F. fructosus*, and *Z. rouxii* within the tested SL concentration range, with a MLC of 15 and 8 g·L^−1^ di-acetylated lactonic SLs for *S. aureus* and *Z. rouxii*, respectively, and a MLC value of the 10 g·L^−1^ wild-type SL mixture for *F. fructosus*. For all tested microorganisms, at least one MIC value could be determined within the tested range of one of the compounds. *S. bombicola* on the other hand was not inhibited by the addition of SLs within the tested range, granting it a competitive advantage, which thus indeed confirms this to be a plausible natural function or advantage of SLs for *S. bombicola*.

### 3.2. Protection against Osmotic Pressure

Hommel et al. (1994) suggested that SLs produced by *S. apicola* aid in the adaptation of the yeast to high osmotic pressure, arising from the prevalence of sugars in honey and nectar, characterizing its natural environment (see Table 1). They noted parallels in the biosynthesis of the sophorose moiety of SLs and trehalose synthesis in *S. cerevisiae* [38]. The latter is known to act as a compatible solute under (osmotic) stress conditions [59]. However, compatible solutes are defined as highly water soluble and low molecular mass molecules that accumulate to high intracellular concentrations, three conditions that are not fulfilled for SLs [60]. Inverting this rationale, one could theoretically imply a relief of osmotic stress: the osmotically active, highly soluble, low molecular weight sugars outside the cell are converted into high molecular weight and less soluble SLs, thereby alleviating the osmotic pressure on the microbial cell. If this process represents (one of) the natural function(s) of SLs, it should entail enhanced growth of a strain capable of making SLs compared to a strain deficient in SL biosynthesis when grown on high sugar concentrations, which will be investigated here.

**Figure 3:**
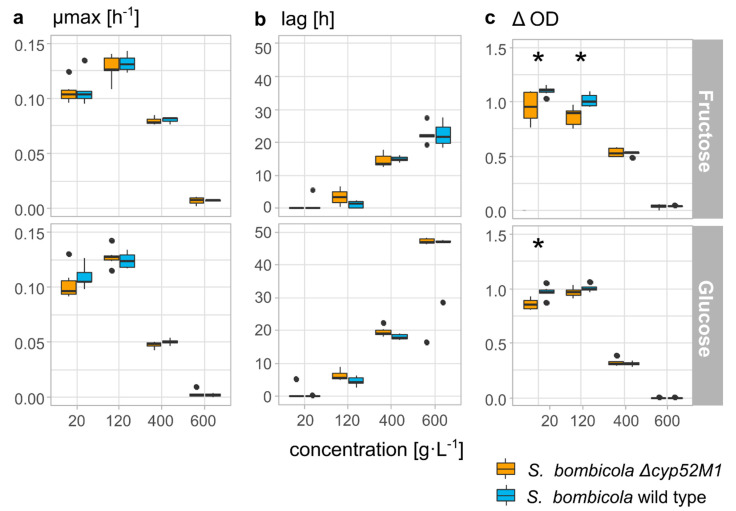
Boxplots of the growth parameters of a sophorolipid deficient *S. bombicola* *Δcyp52M1* strain (yellow boxes, left) and a sophorolipid producing *S. bombicola* wild-type strain (blue boxes, right) on increasing concentrations (20 g·L^−1^ to 600 g·L^−1^) of fructose (top) and glucose (bottom) (*n* = 6). (**a**) The maximal growth rate µ_max_ (h^−1^), (**b**) the duration of the lag phase (h), and (**c**) the ΔOD reached during growth (measured OD_600_ value of the stationary phase minus value at t_0_). Significant differences in growth between both strains are marked on top of the corresponding boxes: * 0.01 < *p* ≤ 0.05.

To evaluate if sophorolipids (SLs) aid *S. bombicola* to withstand osmotic pressure caused by the prevalence of sugars in its natural environment (mainly fructose, glucose, and sucrose), a growth experiment was carried out with the SL deficient *Δcyp52M1 S. bombicola* strain and the wild-type *S. bombicola* strain on increasing concentrations (up to 600 g·L^−1^) of glucose or fructose. The results of the determined growth parameters (maximum growth rate µ_max_ (h^−1^), the duration of the lag phase (h), and the ΔOD) are depicted in Figure 3. There were no significant differences observed in the growth profiles and parameters between the SL deficient *S. bombicola* *Δcyp52M1* strain and the wild-type *S. bombicola* strain, except for the ΔOD values obtained on 20 g·L^−1^ fructose and glucose, and 120 g·L^−1^ fructose, with p-values of 0.04, 0.02, and 0.02, respectively. For the latter cases, the wild type was able to reach slightly higher OD values/cell densities, suggesting a small fitness cost of the knockout of the *cyp52M1* gene. An analysis of the end cultures (t = 60 h) grown on 120 g·L^−1^ glucose or fructose confirmed de novo sophorolipid production for the wild-type strain in contrast to a lack of SL production in the *S. bombicola* *Δcyp52M1* strain (see Figure S1). However, this production of SLs did not result in better growth in comparison to the condition where they were not produced, as growth declined for both strains when sugar concentrations increased with almost no growth observed on 600 g·L^−1^ of sugar. Interestingly, fructose seems to give rise to better growth than glucose, especially at higher concentrations, which corresponds with the fact that *S. bombicola* is a fructophilic yeast (preference for fructose over glucose) [61]. Microscopic observations of the end cultures also showed no aberrant morphological differences between a wild-type and a *Δcyp52M1* strain, nor between low or high sugar concentrations (see Figure S2). At 400 and 600 g·L^−1^ of glucose or fructose, distinctly less to no cells were visible, corresponding to the lower OD values which were therefore not caused by cell shrinkage. Similar growth profiles were noticed when sucrose was used as the carbon source when molecular masses are considered (e.g., the growth curve on 800 g·L^−1^ of sucrose coincides with the one on 400 g·L^−1^ of glucose; see Figure S3). This could be expected as osmotic pressure is a colligative property, which means it only depends on the amount of dissolved solute molecules. These results indicate that the capability of producing SLs does not entail protection against high osmotic pressure, at least not under lab conditions.

### 3.3. Uptake of Hydrophobic Substrates

In early research, the production and secretion of SLs (and biosurfactants in general) were described in relation to the growth on hydrophobic carbon sources and were considered as a means for the producing organisms to effectuate the catabolization of hydrophobic substrates such as alkanes, whether or not through their emulsifying properties [39,40]. However, Hommel et al. later stated that the biosynthesis of SLs by *Starmerella* yeasts cannot simply be a prerequisite for the degradation of extracellular hydrocarbons, as SL production based on glucose (and other sugars) alone was reported for *S. apicola* and *S. bombicola*, independent of the fact that hydrocarbons were present or not [38,62]. Moreover, the evaluated alkanes in the literature in this respect are not typically present in the natural habitat of *S. bombicola* where SL biosynthesis has evolved. Therefore, two types of natural accessible hydrophobic substrates present in the habitat of *S. bombicola* were evaluated in this study: beeswax and triacylglycerols of rapeseed oil (plant oil), alongside hexadecane as a control from the literature.

To examine if the presence of SLs enhances the catabolization of these hydrophobic substrates, most likely through solubilization/emulsification effects exerted by SLs, a growth experiment was performed with 20 g·L^−1^ of beeswax, rapeseed oil, or hexadecane as the sole carbon source. Both a SL producing wild-type and a SL deficient *Δcyp52M1* strain [47] were evaluated in the absence and presence of 1 g·L^−1^ of the wild-type SLs mixture, and several growth parameters were estimated: the estimated growth rate µ (h^−1^), the duration of the lag phase (d), and the ΔOD (corresponding to the produced biomass). The cells were cultured in glass tubes because of the inability to grow on rapeseed oil in deep well plates (a substrate that certainly can be metabolized by a wild-type *S. bombicola*), probably due to altered oxygen availability. Consequently, it was hard to keep the cultures sterile for longer than 7 days when growth was lagging. Therefore, growth parameters were calculated based on OD measurements up to day 7 for beeswax and hexadecane, and up to day 14 for the inoculated cultures on rapeseed oil. No growth was observed for the inoculated negative controls (on the medium without a C-source, with and without the addition of SLs) for at least 7 days.

**Figure 4:**
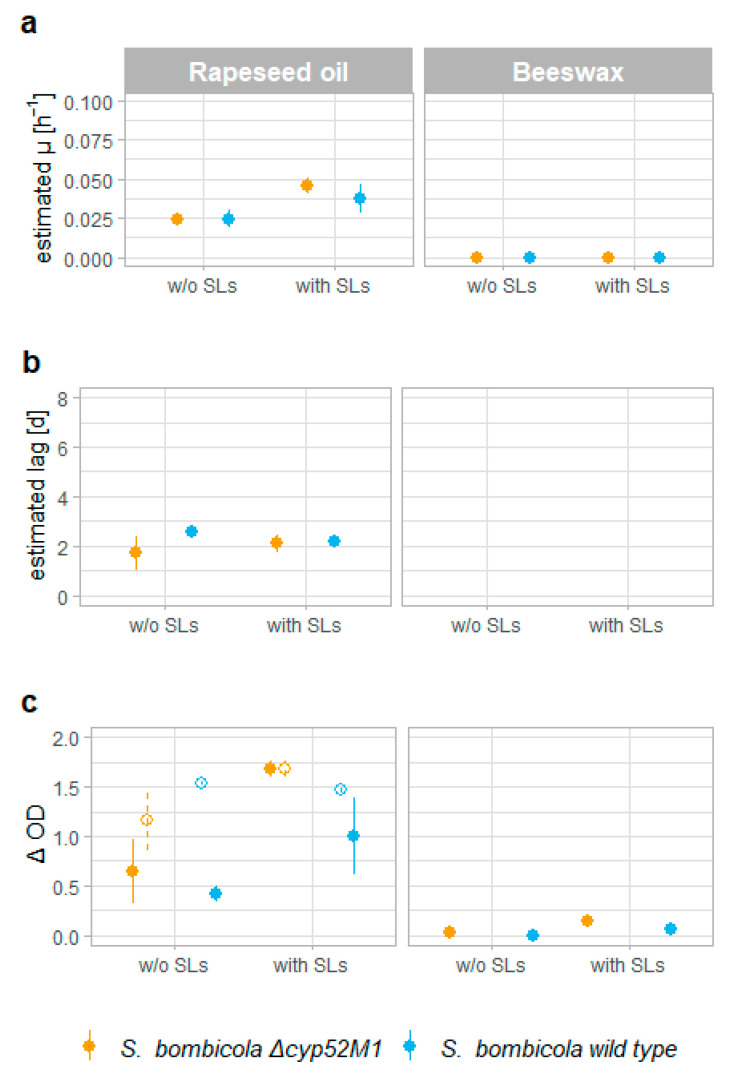
Scatterplots of the estimated growth parameters of a sophorolipid deficient *S. bombicola* *Δcyp52M1* strain (yellow dots, left) and a sophorolipid producing *S. bombicola* wild-type strain (blue dots, right) on rapeseed oil (*n* = 3) and beeswax (*n* = 2) in the presence (‘with’) or absence (‘*w*/*o*’) of 1 g·L^−1^ wild-type sophorolipid mixture. The depicted error bars represent the standard deviation. (**a**) The estimated growth rate µ (h^−1^), (**b**) the estimated duration of the lag phase (d) (cultures that lagged until the end of the experiment are not depicted on the plot), and (**c**) the ΔOD (maximum measured OD_600_ value minus value at t_0_) at day 7 (full dots) and for rapeseed oil also at day 14 (empty dots).

For beeswax—a solid substrate consisting of a complex mixture of long chain monoesters (C40–C48), hydrocarbons (C27–C33), and fatty acids (C24–C32)—no growth was visible after 7 days for either of the two *S. bombicola* strains, neither in the presence or absence of SLs (Figure 4). *S. bombicola* is not able to use beeswax as a sole carbon source (at least not under submerged lab conditions after 7 days of incubation).

On the other hand, both the wild-type and the *Δcyp52M1 S. bombicola* strains were able to grow on rapeseed oil—consisting of triacylglycerols of mainly oleic, linoleic, and linolenic acid—implying that SLs are no prerequisite for the catabolization of rapeseed oil. The addition of 1 g·L^−1^ of SLs did not decrease the duration of the lag phase (Figure 4b), but slightly increased the growth rate (Figure 4a), although this difference is only significant for the *Δcyp52M1* strain: *p* = 0.03 vs. *p* = 0.14 for the wild type. One could argue that this observation could be caused by the fact that SLs are also produced by the wild type (SL production was confirmed at day 14; see Figure S4) and hence already promote growth without the additional supplemented SLs. Nevertheless, the estimated µ of the wild type was not higher than the µ of the *Δcyp52M1* strain, and the presence of SLs in the culture broth could not be confirmed yet after 7 days (TLC, see Figure S4). Improving the statistical power by increasing the sample size might help if there was an undetected significant difference for the wild type (with and without the addition of SLs) in this experiment. The higher growth rates for the cultures with added SLs are also reflected in the ΔOD values (equaling the total grown biomass) at day 7, with a *p* value of 0.03 for the *Δcyp52M1* strain and 0.09 for the wild type (see Figure 4c). The cultures with added SLs already reached the stationary phase after 7 to 9 days, while some of the cultures without SLs did not even reach the stationary phase after 14 days (see Figure S5).

These observations support the hypothesis that SLs can promote growth on some hydrophobic substrates, in this case rapeseed oil, probably through a solubilization effect. This theory however cannot be generalized to all hydrophobic substrates: no growth was observed after 7 days on hexadecane as the sole C-source when SLs were added for both the wild-type and the *Δcyp52M1 S. bombicola* strains, although without SLs the estimated growth rates µ were higher than for rapeseed oil (see Figure S6). Additionally, the ability of producing SLs is clearly not sufficient to promote growth on rapeseed oil directly, as the growth of the wild type is not surpassing that of the SL deficient *Δcyp52M1* strain (in both µ, lag, and ΔOD) in the first 7 days; only when SLs were already present/added in the pre-exponential phase a beneficial effect was noticed, indicating that growth on hydrophobic substrates is a less important physiological function of SLs.

### 3.4. Exclusive Storage Compound

The possibility that one of the functions of SLs could be the formation of an extracellular storage compound has long been underexposed. However, converting an easily accessible carbon source such as glucose or fructose (present in honey and nectar) into an energy-rich yet metabolically less accessible molecule such as sophorolipids is an excellent mechanism to compete with other organisms populating the same habitat. In this way, the organism can monopolize the carbon source present in the environment and simultaneously build up an energy reserve that can be utilized later on. This function of SLs was first theoretically postulated by Hommel et al. (1994)*,* as trehalose not only functions as a compatible solute in *S. cerevisiae* (see above) but also acts as a storage compound [38,63]. This theory thus implies that *S. bombicola* should be able to catabolize its own SLs. This was indeed suggested by Garcia-Ochoa et al. (1996) who claimed that the SL concentration decreased from 5 to 1 g·L^−1^ when used as the sole carbon source, although later on other authors claimed that *S. bombicola* is not able to metabolize SLs [64,65,66].

The idea that SLs—categorized as secondary metabolites—could serve as an extracellular storage compound is emboldened by similar indications and/or proof for other biosurfactants/glycolipids: cellobiose lipids [67], surfactin [68], and mannosylerythritol lipids (MELs) [69]. Moreover, this theory also holds for *Pseudohyphozyma bogoriensis* as the disappearance of its branched SLs from an old culture medium was already reported in 1961 [70]. The possible catabolization of SLs by the producing organism *S. bombicola* will be investigated and discussed in detail in this research article.

#### 3.4.1. Growth on Production Medium

**Figure 5:**
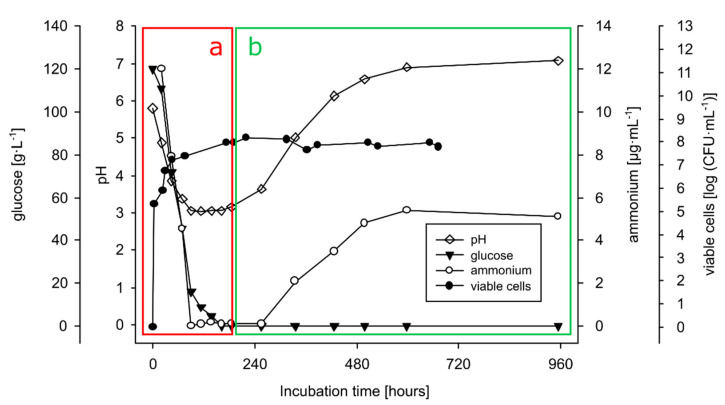
(**a**) SL biosynthesis and (**b**) SL catabolization phase of *Starmerella bombicola* cultivated on the production medium. (◊) pH, (▼) glucose concentrations, (○) ammonium concentrations, and (●) viable cell concentrations are shown.

**Figure 6:**
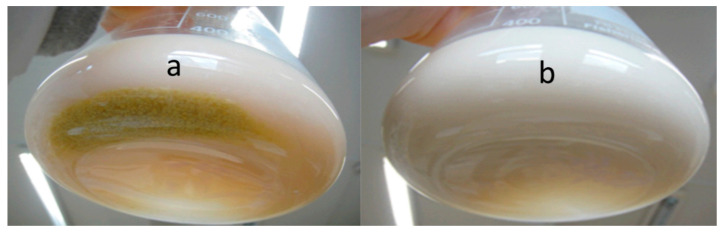
Shake flask culture of *Starmerella bombicola* cultivated on the production medium after (**a**) 240 h (10 days) of cultivation and (**b**) 960 h (40 days) of cultivation.

**Figure 7:**
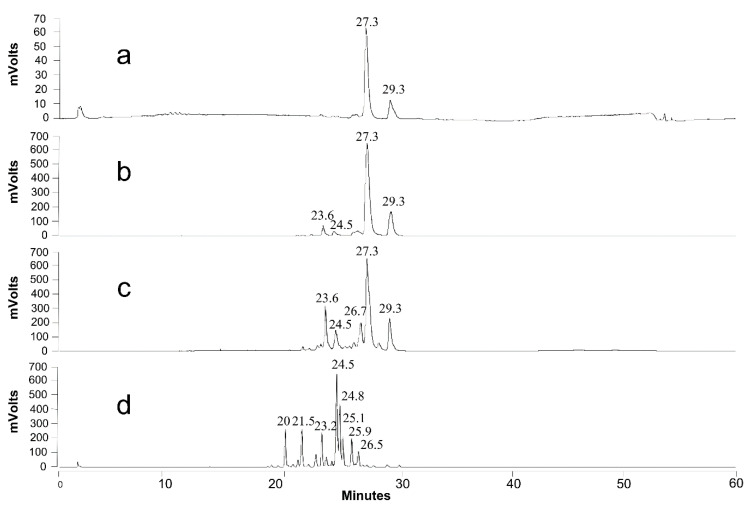
HPLC-ELSD chromatograms from a *S. bombicola* culture on production medium taken after (**a**) 8 days, (**b**) 15 days, (**c**) 40 days, (**d**) and 100 days of incubation. Peaks correspond to di-acetylated lactonic SLs C18:0 (29 min) and C18:1 (27 min), mono-acetylated lactonic SLs (23 and 24 min), di-acetylated acidic SLs (23 and 24 min), mono-acetylated acidic SLs C18:1 (21 min), non-acetylated acids SLs C18:1 (20 min), and hydroxylated fatty acids (25 and 26 min).

The theory that sophorolipids (SLs) constitute a build-up of carbon and energy in *S. bombicola* denotes that this yeast should be able to catabolize its own produced SLs. To investigate this, a prolonged combined production and catabolization experiment was performed: *S. bombicola* was grown on the SL production medium described by Lang (Table 2) (rapeseed oil was added after 48 h), and the cultures (*n* = 3) were incubated beyond glucose exhaustion (for 100 days) to follow up typical parameters and to evaluate potential evidence for SL catabolization. The results are shown in Figure 5, Figure 6 and Figure 7.

The first phase of the experiment (Figure 5a) can be distinguished as the SL production phase: di-acetylated lactonic SLs (C18:1 and C18:0) were the predominantly produced forms of SLs as shown in Figure 7a. Lactonic SLs are typically the dominant form of SLs produced by *S. bombicola* on the production medium and start to precipitate after 130 h (±5.5 days) (see Figure 6a) because their solubility dramatically decreases at lower pH values (see Table S1). The observed strong pH decrease (see Figure 5a) resulted from the secretion of organic acids such as citrate and isocitrate (up to 10 g·L^−1^) and the utilization of the nitrogen source during cell growth [71].

The second phase of the experiment (see Figure 5b) is initiated upon glucose depletion at about 10 days of cultivation. A steep pH and ammonium rise were observed. The pH rise can (partly) be explained by the increase of free ammonium ions, which is the result of the deamination of amino acids once glucose is depleted and the yeast shifts from carbohydrates to proteins as the energy source. The produced organic acids can also be used as a carbon source as the concentrations of citrate and isocitrate were reported to decrease when the glucose in the culture medium was nearly consumed [72]. Remarkably, the precipitated SLs (di-acetylated lactonic form) started to disappear again after 330 h (±14 days) of incubation to vanish eventually completely (see Figure 6b).

Although a rising pH promotes the solubilization of the precipitated SLs, a LC-MS analysis also showed that the SL mixture became enriched in more hydrophilic compounds: after 15 days, mono-acetylated lactonic SLs (24 min) and di-acetylated acidic SLs (23 min) appeared (Figure 7b), and their concentration increased with time (Figure 7c: 40 days). After 100 days of cultivation (Figure 7d), di-acetylated lactonic SLs nearly completely disappeared (peak at 27 and 29 min for C18:1 and C18:0, respectively) and a complex mixture of compounds was present in the extracellular medium: mono- and di-acetylated lactonic SLs (still present in minor amounts); di-, mono-, and non-acetylated acidic SLs; (acetylated) glucolipids and hydroxylated fatty acids. Remarkably, the log CFU values, a marker for cell viability, remained constant (as shown in Figure 5) for over 100 days without the substrate addition (log CFU values at 960 h could not be determined due to contamination on the dilution plates; log CFU values at day 100 are not shown). These observations thus clearly indicate that *S. bombicola* can catabolize SLs in times of starvation. However, it is obviously plausible that the incubation of solubilized SLs in the watery medium environment at 30 °C and neutral pH values for over 3 months could give rise to spontaneous hydrolysis. Especially ester functionalities (acetyl- and lacton functions) are quite prone to spontaneous hydrolysis in water, particularly at pH values above 6. This thus required further investigation.

#### 3.4.2. Growth on Sophorolipids as the Sole Carbon Source

If the disappearance of sophorolipids (SLs) during prolonged incubation as described above is caused by SL catabolization, *S. bombicola* should be able to grow on the medium with SLs as the sole carbon source (called the SL medium). A specific cultivation experiment to investigate this was set up as described under the methods and materials section. After three to four days of cultivation, multipolar budding of *S. bombicola* was indeed observed, although high cell densities were not attained. The observed growth could clearly be linked to the consumption of the added SLs by *S. bombicola*, as after 1 month of cultivation, almost all the SLs and derivatives disappeared from the extracellular culture medium (Figure S7d and Table S2).

**Figure 8:**
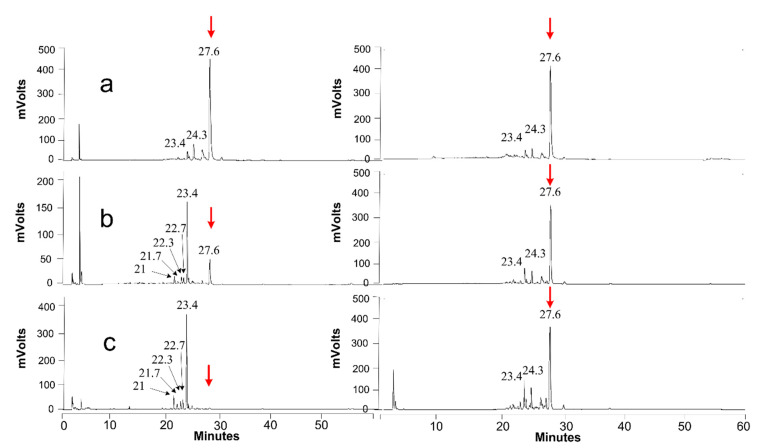
(**left**) HPLC-ELSD chromatograms of samples from the extracellular activity assay (unconcentrated secretome) incubated with di-acetylated lactonic SLs (27.6 min) after (**a**) 1 h, (**b**) 10 days, and (**c**) 32 days of incubation; (**right**) controls for SLs at each time point. Peaks at 23.4 min = co-eluting di-acetylated acidic SLs and mono-acetylated lactonic SLs; other peaks at 20–23 min = mono- and non-acetylated acidic SLs.

Similar to the observations of the first experiment described above, the first two catabolic activities that were detected were deacetylation and the ring-opening of the SLs resulting in mono-acetylated lactonic SLs and di-acetylated acidic SLs, respectively (Figure S7a). Longer cultivation gave rise to a further breakdown of SLs into mono- and non-acetylated acidic SLs, and minor amounts of non-acetylated lactonic SLs (C18:1) were also detected (Figure S7b). After 30 days of cultivation, almost all the SLs disappeared from the culture medium (Figure S7d). Importantly, near to no hydrolysis of SLs was observed in the controls (incubation of the cell free SL medium) (similarly as shown in Figure 8, right). The pH in the cultures only dropped from 6.06 to 5.60 during cultivation (after 16 days), so spontaneous hydrolysis of the ester functionalities, which can occur at higher and very low pH values, can also not be argued.

These results thus not only affirm the catabolization of SLs by *S. bombicola*, but also suggest the presence of extracellular enzyme(s) responsible for part of these catabolic processes: one or more hydrolyzing enzymes responsible for the ring-opening of the lactonic SLs and deacetylation of lactonic and acidic SLs. Moreover, in contrast to the first experiment, no glucolipids, hydroxylated fatty acids (nor glucose or sophorose) were detected in the culture medium. This suggests that non-acetylated acidic SLs are taken up by the starved cells to be further metabolized intracellularly.

#### 3.4.3. Extracellular Activity (of the Secretome)

To further investigate the catabolic steps, unconcentrated secretomes (*n* = 3) were prepared (from 21-day-old production cultures) and incubated with di-acetylated lactonic or non-acetylated acidic SLs.

For di-acetylated lactonic SLs, extracellular conversion into di-acetylated acidic and mono-acetylated lactonic SLs was detected after 10 days (both compounds co-eluted at 23.4 min; see Figure 8b). The conversion was complete after 32 days of incubation (see Figure 8c). The peaks appearing between 20 and 23 min correspond to mono- and non-acetylated acidic SLs. Some ring-opening and deacetylation was also observed for the control incubation of SLs (see Figure 8, right panels), but these spontaneous effects were clearly a lot less pronounced than those for the incubation with unconcentrated secretomes (the pH remained stable at about 5.5 for all the assays and controls).

**Figure 9:**
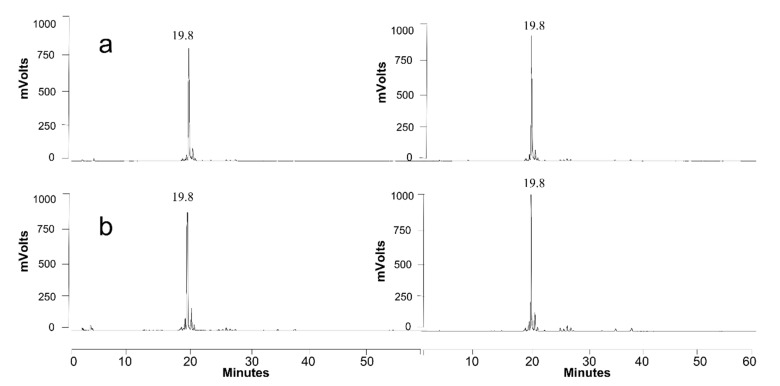
(**left**) HPLC-ELSD chromatograms of samples from the extracellular activity assay (unconcentrated secretome) incubated with non-acetylated acidic SLs (19.8 min) after (**a**) 1 h and (**b**) 16 days of incubation; (**right**) controls for SLs at each time point.

For non-acetylated acidic SLs, no extracellular conversion was observed (see Figure 9). This indicates that these non-acetylated acidic molecules are the end-products of the extracellular catabolization of SLs by *S. bombicola*. They are thus hypothesized to be taken up again by the starved cells to be further catabolized intracellularly, explaining their disappearance from cultures cultivated on SLs as the sole carbon source (see Figure S7d).

The obtained results were confirmed by repeating these experiments. In addition, assays with concentrated and standardized protein solutions (secretomes, pH set on 7) resulted in similar observations. Moreover, when using concentrated secretomes derived from a culture cultivated on SLs as the sole C-source, the demonstrated activity was a lot higher: all of the added substrate (di-acetylated lactonic SLs) was converted into di-acetylated acidic SLs and mono-acetylated lactonic SLs after only 6 days of incubation (data not shown). This indicates the upregulation of the responsible gene(s) as the used protein concentrations were constant.

A first ‘suspect’ for the ring-opening of lactonic SLs that the authors postulated was the *S. bombicola* lacton esterase enzyme [48]. The *sble* gene encodes for the enzyme responsible for the lactonization of SLs during the biosynthesis of SLs and could thus be responsible for the opposite reaction as well. To investigate this hypothesis, an additional unconcentrated secretome was prepared of the Δ*sble* strain [49] to investigate if ring-opening still occurred. As ring-opening was still observed after 32 days of incubation in a SBLE free secretome, it can be concluded that the SBLE enzyme is not the (only) enzyme responsible for ring-opening (see Figure S8).

#### 3.4.4. Intracellular Activity (of Cell Lysate)

To investigate the intracellular degradation of SLs, lysates of wild-type cultures were prepared (from 21-day-old production cultures, *n* = 3). The assays were incubated for 6 days for di-acetylated lactonic SLs and 9 days for non-acetylated acidic SLs.

Incubation of the lysates with lactonic SLs (Figure S9 and Table S3) gave rise to very similar results as those obtained for the cultivation of *S. bombicola* on SLs as the sole C-source: ring-opening and deacetylation were observed. All these intermediates already appeared after only 24 h of incubation, but the effects became more pronounced upon longer incubation. All but one: the emergence of the non-acetylated acidic SLs became only evident after 6 days of incubation. Further disassembly of SLs was not detected, possibly because the incubation time (6 days) was not long enough to allow the build-up of non-acetylated acidic SLs (peak at 19.6 min) of which catabolic intermediates could subsequently be derived and detected.

**Figure 10:**
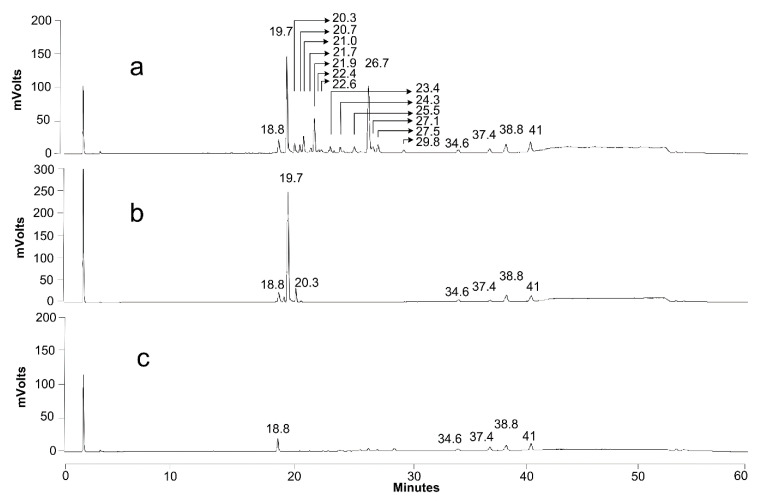
HPLC-ELSD chromatograms of (**a**) an enzyme assay for intracellular activity (lysate) on non-acetylated acidic SLs (peak at 19.7 min) after 9 days of incubation (**b**,**c**) correspond to the controls for SLs and the lysate, respectively. The peak at 18.8 min corresponds to an internal standard (C12:0-OH).

Incubation of the lysate with the non-acetylated acidic SLs (19.7 min) for 9 days leads to clear degradation: the biggest peaks appearing correspond to C18:1 glucolipids (21.9 min) and C18:1 hydroxylated fatty acids (26.7 min). Other compounds include other hydroxylated fatty acids derived from other SL variants present in the substrate, giving rise to C18:0, C18:2, and C16:0 hydroxylated fatty acids (Figure 10a; and Table 3). These effects were only visible after 6 days of incubation, indicating a slower/less efficient process or lower enzyme concentrations compared to the one described above for di-acetylated lactonic SLs (activity detected after 24 h at the same total protein concentrations). Together, these results give a strong indication that the catabolization of non-acetylated acidic SLs predominantly occurs intracellularly. None of the abovementioned effects were observed when repeating this experiment with a lysate from a culture that was cultivated for only 4 days (glucose not depleted), so the responsible enzymes are probably not expressed yet.

Non-acetylated acidic SLs are thus disassembled intracellularly into glucolipids and hydroxylated fatty acids, suggesting that at least a stepwise detachment of the glucose molecules is occurring. This was confirmed by measuring the glucose concentrations of the end samples (after 9 days of incubation). In contrast, no glucose was detected in the controls, so the glucose released in the assay was clearly derived from hydrolyzed SLs. However, this result does not rule out the possibility that the sophorose molecule can (also) be released, followed by hydrolysis of the disaccharide into two glucose molecules; indeed, minor amounts of a compound corresponding to the mass of sophorose were detected by performing HPLC-MS (data not shown).

One last remarkable observation was that incubation of cell lysates with non-acetylated acidic SLs also resulted in the formation of acetylated intermediates (glucolipids and acidic SLs) (Table 3). These compounds were not present in the controls, and can hence only arise from the action of the acetyltransferase responsible for the acetylation of SLs [73]. This indicates that this enzyme must still be active in the cell lysates of cultures that were cultivated for 21 days. This acetylation was already detected after only 24 h of incubation, indicating a more efficient biosynthetic process as compared to the catabolic one.

## 4. Discussion

Although potential applications for sophorolipids (SLs) are currently expanding, the physiological role of these SLs for their producing organism *Starmerella bombicola* remained speculative. Based on their properties, biosynthesis, and some sparse supporting data, five hypotheses could be found scattered around the literature, of which four were investigated in this research article. According to the results presented here, one hypothesis can be discarded: SL production as a protection against osmotic pressure. The other hypothesized natural functions—antimicrobial activity, uptake of hydrophobic substrates, and exclusive storage compound—are supported by our findings.

### 4.1. Antimicrobial Activity

The antimicrobial activity of SLs was already described before. However, varying MIC values are reported for wild-type SL mixtures or one of its components: e.g., for wild-type SL mixtures, MICs are reported of 0.5–10 g·L^−1^ for *E. coli* and of 0.3–4.5 g·L^−1^ for *S. aureus* [74,75]; and for C18:1 non-acetylated acidic SLs and *P. aeruginosa*, a MLC of 5 g·L^−1^ was reported, while a MIC of 10 g·L^−1^ was found in this study (Figure 2) [15]. These differences can be partly explained by different compositions of the SL mixture (i.e., fatty acid chain lengths, acetylation, and/or lactonization degree and purity), different assay methods, or different tested isolates. However, the determinants defining the antimicrobial activity could be more complex, as Haque et al. found a MIC of 0.06 g·L^−1^ for di-acetylated lactonic SLs against *C. albicans* in comparison to the 7.7 g·L^−1^ found in this research paper, using the same isolate and assay but a different medium [36]. Moreover, the MIC value of the wild-type SL mixture for *F. fructosus* is lower than the MICs of its comprising compounds, suggesting a possible synergistic effect.The natural habitat of *S. bombicola*, i.e., flowers and (bumble)bees, harbors a wide variety of microorganisms [52,53,54,55,56,57]. Proving that several tested genera, next to three ecologically highly relevant species (*H. alvei, F. fructosus*, and *Z. rouxii*) are inhibited by SLs correlates with the broad antimicrobial activity that is needed to favor *S. bombicola*—showing a clear competitive advantage over the other tested microorganisms, as it was not inhibited by 20 or 30 g·L^−1^ of SLs—in these environments (see Figure 2). SLs presumably exert their antimicrobial activity by changing or rupturing cellular membranes and hence target a fundamental and universal prerequisite for the survival of cells [76,77]. How *S. bombicola* acquired an increased resistance and circumvents this antimicrobial mechanism is not clear.

Finally, the fact that SLs are regarded as secondary metabolites also fits with the theory that *S. bombicola* produces SLs for their antimicrobial activity. Nevertheless, the SL production titers that *S. bombicola* reaches in lab conditions greatly exceed the inhibitory concentrations, which are in turn some orders of magnitude higher than MIC values of ‘true antibiotics’ (such as penicillin or kanamycin) implying that antimicrobial activity cannot be the sole/main physiological function of SLs [78].

### 4.2. Protection against Osmotic Pressure

When growth of a SL producing wild-type *S. bombicola* strain and a SL deficient *S. bombicola* *Δcyp52M1* strain was compared on glucose and fructose concentrations of 400 or 600 g·L^−1^, no significant differences in growth curves (neither in lag, µ_max_, or ΔOD) were noticed. Both strains showed the fastest growth (highest µ_max_) on 120 g·L^−1^ sugar, with slightly higher OD values for the wild-type strain, indicating a small fitness cost of the knockout of the *cyp52M1* gene. However, these small differences are no longer prevalent for higher sugar concentrations, i.e., almost no growth remaining at 600 g·L^−1^. The hypothesis that the production of SLs was selected for due to the protection of *S. bombicola* by SLs against high osmotic pressure can thus be rejected.

### 4.3. Uptake of Hydrophobic Substrates

SLs are classified as biosurfactants and are known for their emulsifying properties which could enhance contact with and the subsequent uptake of water-insoluble hydrophobic substrates (e.g., applied in bioremediation). The only supporting data for this hypothesis as the natural function of SLs are provided by Ito et al., mentioning a shortened lag phase on hexadecane when 0.4 g·L^−1^ wild-type SLs were added to the medium [39]. Contrary results were obtained in this study: the addition of 1 g·L^−1^ SLs halted the little growth that was observed in the cultures without the SL addition (Figure S6). It is not clear if this discrepancy is caused by a different SL composition (Ito et al. claimed that mono- and di-acetylated lactonic SLs were not responsible for growth stimulation) or a higher added SL concentration. If SLs indeed improve the availability of hexadecane to the cells and the latter would be toxic at a certain concentration, too high SL concentrations might be detrimental.

SLs could not induce growth on beeswax, which corresponds to the fact that Detry et al. could not isolate yeasts from surface swabs of empty wax cells [79]. However, SLs are able to increase the growth rate on rapeseed oil significantly for a SL deficient *Δcyp52M1* strain (but are no prerequisite for growth) and hence might confer their producing microorganism with an evolutionary benefit (Figure 4). It is not clear if this growth benefit applies to other hydrophobic substrates that can be catabolized by *S. bombicola*. This should be further investigated besides the mechanism for this enhanced growth in the presence of SLs, which is probably the promoted uptake of the hydrophobic substrate through emulsification. This enhanced uptake of substrates such as triglycerides can in turn reinforce the SL production as they can be incorporated in newly produced SLs and, as such, invigorate additional competitive advantages of SLs (as described below).

However, the presence of hydrophobic substrates does not (directly) ‘induce’ SL production: for growth on rapeseed oil, a SL producing wild type did not show an advantage over a non-SL producing Δ*cyp52M1* strain when no SLs were added, and SLs could not be detected in the culture broth of the wild type at 7 days of incubation. Moreover, SLs are also synthesized when only glucose is present in the medium (see Figure S1). These observations suggest that the improved uptake of hydrophobic substrates may be a less important natural function of SLs.

### 4.4. Exclusive Storage Compound

Lastly, the catabolization of SLs by *S. bombicola* was also investigated. SLs disappeared from the production medium during prolonged incubation and *S. bombicola* is able to grow on SLs as the sole C-source and can thus effectuate catabolization of its own glycolipids in contrast to what was stated previously [66]. This catabolization consists of the action of secreted enzymes at least responsible for the ring-opening of (di-acetylated) lactonic SLs and deacetylation of acetylated acidic and lactonic SLs. These extracellular processes eventually give rise to non-acetylated acidic SLs.

This theory also holds for *P. bogoriensis*, as the disappearance of its branched SLs from old culture medium was already reported in 1961 [70]. In agreement with our findings, the gradual disappearance of di-acetylated SLs occurred after 3.5 days of cultivation of *P. bogoriensis*, and the simultaneous appearance of mono- and non-acetylated derivatives was observed [80]. An acetylesterase capable of performing these deacetylation reactions in *P. bogoriensis* was identified some years later [81].

Incubation of non-acetylated acidic SLs with the unconcentrated secretomes of *S. bombicola* did not lead to the formation of glucolipids and/or fatty acids, so acetylation does not seem to be a protective mechanism against attack by an extracellular glycosylhydrolase-like enzyme, which is the case for the cellobiose lipid biosurfactants produced by *P. flocculosa* (flocculosin): deac(et)ylation of the glycolipids at high pH values leads to a very fast extracellular metabolization of the deac(et)ylated derivatives [67]. This indicates that these non-acetylated acidic molecules are the end products of the extracellular catabolization of SLs by *S. bombicola*. They are hypothesized to be taken up again by the starved cells—which requires the presence of a SL transporter other than the MDR transporter, as this is an ABC transporter, and these are exclusively reported to show export activity—to be further catabolized intracellularly, explaining their disappearance of cultures cultivated on SLs as the sole carbon source. However, acetylation could still be a protective mechanism to prevent the uptake of acetylated SL derivatives, as was suggested for other types of molecules by Danchin [82].

Further catabolization of non-acetylated acidic SLs by hydrolysis of the glycosidic linkage between the two glucose moieties and between the sophorose- and fatty acid moiety was shown to happen (predominantly) intracellularly. For SL catabolization, at least a stepwise removal of the glucose molecules exists, as both glucolipids and hydroxylated fatty acids were detected in the assays. Whereas Garcia-Ochoa et al. (1996) reported the substantial release of sophorose from SLs, we did not share this observation, as sophorose was only detected in very small amounts [64]. However, the possible enzymatic cleavage of the released sophorose could lead to an underestimation of the total release of this disaccharide in the assays. For comparison: for flocculosin (cellobiose lipid), glucose was shown to be released by catabolic enzymes, whereas cellobiose release was never detected [67].

The major catabolic route for *S. bombicola* is thus suggested to consist of extracellular ring-opening and deacetylation, eventually leading to non-acetylated acidic SLs. These molecules are subsequently taken up by the starved cells and further catabolized intracellularly into glucolipids, hydroxylated fatty acids, sophorose, and glucose. This is an effective strategy to protect the extracellular storage material (especially with antimicrobial activity) against other competing microorganisms, leaving the carbon ‘secured’. Complete and efficient extracellular degradation of deacetylated acidic SLs would lead to the release of glucose and fatty acids in the extracellular space, also accessible to other (competing) microorganisms.

### 4.5. SLs and Their Possible Relation to Overwintering

New ensuing questions arise: ‘Which genes are responsible for the catabolization of sophorolipids in *S. bombicola*?’ and ‘Why does this yeast claim nutrients and convert them into exclusive storage compounds in an environment characterized by the prevalence of sugars?’. The first question could be solved by performing proteomic studies combined with knocking out the suspected genes. The resulting information could be very useful to improve SL production by eliminating the catabolic pathway. For the second question, we present the following theory.

*S. bombicola,* and in extension the whole *Starmerella* clade, is closely associated with a flower-bee mutualism: a natural environment abundant in sugars, found in nectar and honey [57,83]. However, in temperate climates, these resources evanesce in winter when flowers and pollinators disappear. Some researchers already have wondered how ‘nectar yeasts’ survive harsh winters: some believe the yeasts overwinter in soil and are redistributed in spring to new budding flowers through wind or crawling insects; others found them to overwinter together with the associated insect [57]. Each species possibly has its own overwintering strategy.

The nectar yeast *Candida bombi,* currently renamed to *Starmerella bombi,* was found to overwinter in the digestive tract of hibernating bumblebee queens [57,84,85]. As this yeast is closely related to *S. bombicola* (the 18S rDNA genes share a 96% sequence identity), it is very reasonable that the same overwintering strategy also holds for the latter.

Moreover, several observations strengthen a close association of bumblebees and *S. bombicola*. Although some easily generalize the habitat of *S. bombicola* to ‘bees and honey’, Spencer et al. mentioned that despite being isolated first from nectar, they never obtained such high numbers as in bumblebee honey (*Bombus* ssp.) [86]. Additionally, *S. bombicola* was only isolated once from honey bees (*A. mellifera*) or its associated products [79]. These bee species differ in some remarkable ways.

First of all, sugar concentrations in the finished honey of *A. mellifera* are around 84%, too high to allow the growth of yeasts. This is also reflected by the data in Figure 3: growth is already strongly repressed at 400 g·L^−1^ and almost completely ceased at 600 g·L^−1^. In addition, honey bee honey is characterized by an equal amount of glucose and fructose. In contrast, bumblebees produce, next to the ‘thick’ concentrated honey with 70–87% sugar, also ‘thin’ honey with sugar concentrations of 42–52% (from this thin honey, Spencer et al. isolated *S. bombicola*). Moreover, bumblebee honey contains much more fructose than glucose, matching the fructophilic nature of *S. bombicola* (see Table 1) [61,87,88].

Lastly, and most importantly, there is a crucial difference in their behavior: honey bees actively survive winter as a colony, thereby relying on their stored honey, whereas for bumblebees, only the new queens overwinter [89,90]. Before wintertime, the new queens accumulate reserves of fat and glycogen. With some last honey in their crop, they leave the old nests and burrow into the soil where they hibernate. When spring arrives, they fly out seeking nectar and meanwhile inoculating new sterile flowers with yeasts. The more yeast cells that are present on the hibernated queen, the more successful they can colonize flowers and subsequently redistribute [91]. Surprisingly, the relative abundance of *S. bombi* seems to increase during hibernation, indicating an improved fitness towards other ‘bumblebee yeasts’ [85]. As hibernating queens are far less metabolically active, and the supply of sugars for the yeasts diminish, an exclusive storage of carbon and energy would be an excellent strategy to keep up cell numbers, preparing the yeast for a head start at early spring. Although no SL production is yet reported for *S. bombi* (which could be due to nonoptimal culture conditions), this exclusive storage compound could be sophorolipids. The same could hold for *S. bombicola*, known to produce excessive amounts of SLs and able to metabolize them steadily (see Figure 5 and Figure 7) to maintain cell numbers to a constant level for over 100 days, which is as long as a winter.

## 5. Conclusions

To summarize, it could not be confirmed that sophorolipids (SLs) protect its producing yeast *S. bombicola* against high osmotic pressure caused by the prevalence of sugars in the yeasts’ natural habitat. On the other hand, wild-type SLs do exhibit antimicrobial activity against a broad range of microorganisms. Furthermore, *S. bombicola* can catabolize its own previously produced SLs: deacetylation and ring-opening occur extracellularly, resulting in non-acetylated acidic SLs which are imported and further degraded intracellularly. On rapeseed oil, increased growth rates were noticed when SLs were added to a *Δcyp52M1 S. bombicola* culture. Further research is needed to confirm the improved uptake through emulsification as the underlying mechanism. However, some observations indicate a less important physiological function of SLs in the growth on hydrophobic substrates. Nevertheless, an improved uptake of the hydrophobic substrate can boost SL production and hence invigorate the natural functions mentioned above.

Based on the results mentioned above, we proclaim that the natural function of SLs in *S. bombicola* is the build-up of an exclusive, extracellular storage compound with inherent antimicrobial activity that can be utilized under starvation conditions. In this way, *S. bombicola* can very efficiently compete for the available carbon and energy sources with other microorganisms populating its habitat; valuable and easily degradable carbon sources, such as sugars and/or fatty acids, are claimed by converting them into more inert compounds: sophorolipids.

## Figures and Tables

**Table 1:** Sugar concentrations (% *w*/*w*) in honey of honey bees (*Apis mellifera*) and bumblebees (*Bombus* sp.) and in nectar of *Berberis darwinii* [25,26].

% *w*/*w*	Honey of *A. mellifera*	Honey of *Bombus* sp.	Nectar of *B. darwinii*
Sucrose	1–3	0.6–3	33.1
Glucose	30–48	5–54	1.2
Fructose	41–54	37–79	0.6
Water (and other)	13.4–26.6	13–30	63.7

**Table 2:** Composition of the used media.

Component (g·L^−1^)	Production Medium ^1,a^	SL Medium ^1,b^	SD CSM Medium ^2^	SD CSM Medium *w*/*o* C-source ^3^
Glucose (Cargill)	120	/	20	/
YNB *w*/*o* AA (BD Difco)	/	4	6.7	6.7
CSM (MP biomedicals)	/	/	0.79	0.79
Yeast extract (Brenntag)	4	/	/	/
3Na-citraat.2H_2_O (Sigma-Aldrich)	5	/	/	/
NH_4_Cl (Sigma-Aldrich)	1.5	1.5	/	/
KH_2_PO_4_ (Sigma-Aldrich)	1	1	/	/
K_2_HPO_4_ (Sigma-Aldrich)	0.16	0.16	/	/
MgSO_4_·7H_2_O (Sigma-Aldrich)	0.7	0.7	/	/
NaCl (Esco)	0.5	0.5	/	/
CaCl_2_·2H_2_O (Merck)	0.27	0.27	/	/
Sophorolipids	/	20	/	/

Used for the experiments in result section: ^1^ Section 3.4. Exclusive storage compound: ^a^ Section 3.4.1. Growth on production medium; and ^b^ Section 3.4.2. Growth on sophorolipids as the sole carbon source. ^2^ Section 3.2. Protection against osmotic pressure.^3^ Section 3.3. Uptake of hydrophobic substrates.

**Table 3:** Identification of the major peaks detected in Figure 10 after HPLC-MS analysis. The detected masses (*m/z*) correspond to the [M − H]^−^ adduct of the identified molecules. The most prominent ones are framed.

Retention Time	*m*/*z*	Identity		Acetylation
18.8	215	hydroxylated fatty acid	C12:0	
19.7	621	acidic SL	C18:1	non
20.3	623	acidic SL	C18:0	non
20.7	663	acidic SL	C18:1	mono
21	459	glucolipid	C18:1	non
21.7	665	acidic SL	C18:0	mono
21.9	459	glucolipid	C18:1	non
22.4	459	glucolipid	C18:1	non
23.4	705	acidic SL	C18:1	di
24.3	501	glucolipid	C18:1	mono
25.5	295	hydroxylated fatty acid	C18:2	
26	271	hydroxylated fatty acid	C16:0	
26.7	297	hydroxylated fatty acid	C18:1	
27.1	299	hydroxylated fatty acid	C18:0	
27.5	297	hydroxylated fatty acid	C18:1	
29.8	299	hydroxylated fatty acid	C18:0	
38.8	255	fatty acid	C16:0	
41	283	fatty acid	C18:0	

## Data Availability

The data presented in this study are available in the results section or in the Supplementary Material of this article.

## Supplementary Materials

The following are available online at https://www.mdpi.com/article/10.3390/jof7110917/s1. Figure S1: UPLC-ELSD chromatograms of the culture broth of the *S. bombicola* wild-type strain and the *S. bombicola Δcyp52M1* strain grown on glucose and fructose. Figure S2: Light microscopic observations of a *S. bombicola* wild-type and a Δ*cyp52M1* strain that were cultivated on 20, 120, and 400 g·L^−1^ fructose or glucose. Figure S3: Growth curves of a *S. bombicola* wild-type and Δ*cyp52M1* strain on medium with glucose or sucrose. Figure S4: TLC analysis of cell culture broths of a *S. bombicola* wild-type and a Δ*cyp52M1* strain on medium with rapeseed oil as the sole carbon source. Figure S5: Growth curves of a *S. bombicola* wild-type and Δ*cyp52M1* strain on medium with rapeseed oil as sole carbon source. Figure S6: Scatterplots of the estimated growth parameters of a sophorolipid deficient *S. bombicola Δcyp52M1* strain and a sophorolipid producing *S. bombicola* wild-type strain on hexadecane. Table S1: Influence of pH on the solubility (g·L^−1^) of different forms of SLs. Figure S7: HPLC-ELSD chromatograms of samples from a *S. bombicola* culture on SL medium with di-acetylated lactonic SLs as the sole carbon source. Table S2: Identification of the major peaks detected in Figure S7 after HPLC-MS analysis. Figure S8: HPLC-ELSD chromatograms of samples from an extracellular activity assay with a S*. bombicola Δsble* strain (unconcentrated secretome) incubated with di-acetylated lactonic SLs. Figure S9: HPLC-ELSD chromatograms of an enzyme assay of the wild-type lysate and di-acetylated lactonic SLs. Table S3: Identification of the major peaks detected in Figure S9 after HPLC-MS analysis.
