# Supplementary material for: Elucidation of the Natural Function of Sophorolipids Produced by Starmerella bombicola
Source: J Fungi (Basel). 2021 Oct 28;7(11):917. doi: 10.3390/jof7110917 (PMC8621470; doi:10.3390/jof7110917)
Supplement: Supplementary file 1 [file jof-07-00917-s001.zip › jof-1430556-supplementary.pdf]

Supplementary information: tables and figures

Elucidation of the Natural Function of Sophorolipids Produced by *Starmerella bombicola*

Veerle De Clercq †, Sophie L. K. W. Roelants *,†, Martijn G. Castelein, Sofie L. De Maeseneire and Wim K. Soetaert

Centre for Industrial Biotechnology and Biocatalysis (InBio.be), Department of Biotechnology, Faculty of Bioscience Engineering, Ghent University, Coupure Links 653, 9000 Ghent, Belgium;
veedcler.DeClercq@UGent.be (V.D.C.); Martijn.Castelein@UGent.be (M.G.C.);
Sofie.DeMaeseneire@UGent.be (S.L.D.M.); Wim.Soetaert@UGent.be (W.K.S.)
* Correspondence: Sophie.Roelants@UGent.be; Tel.: +32-9-264-93-85
† These authors contributed equally to this work.

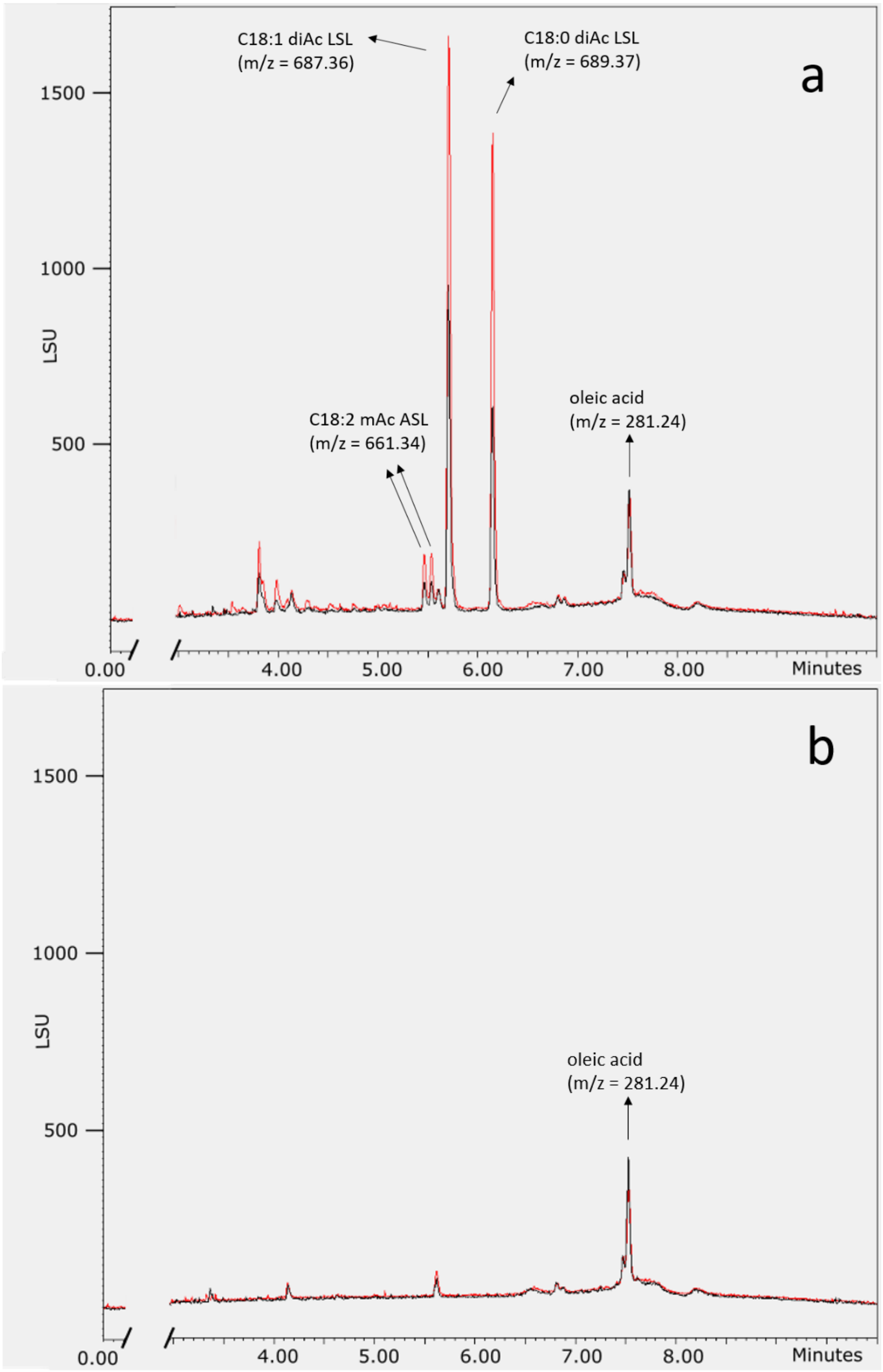

Figure S1. UPLC-ELSD chromatograms of the culture broth after 60 h of cultivation of **(a)** the *S. bombicola* wild-type strain **(b)** and the *S. bombicola Δcyp52M1* strain. The black chromatogram corresponds to the SD CSM medium with 120 $g{\cdot}L^{-1}$ glucose and red chromatogram to the SD CSM medium with 120 $g{\cdot}L^{-1}$ fructose. UPLC-MS was used to detect the masses and identify the corresponding peaks.

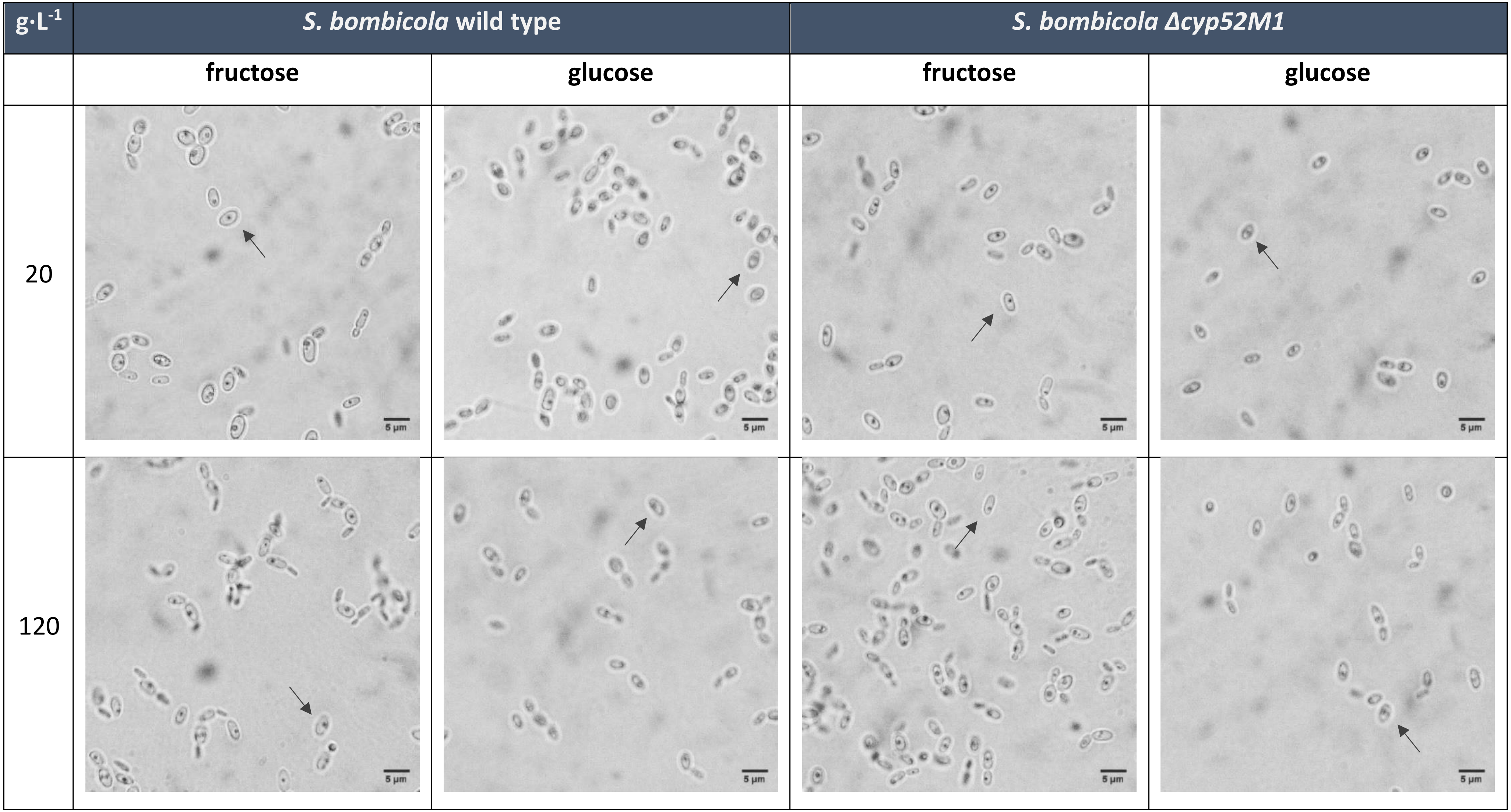

Figure S2. Light microscopic observations of a *S. bombicola* wild-type and a *Δcyp52M1* strain that were cultivated for 60 h on the SD CSM medium with 20, 120 and 400 g·L^{-1} fructose or glucose. The scale bar corresponds to 5 µm and some cells are pointed out with an arrow.

400	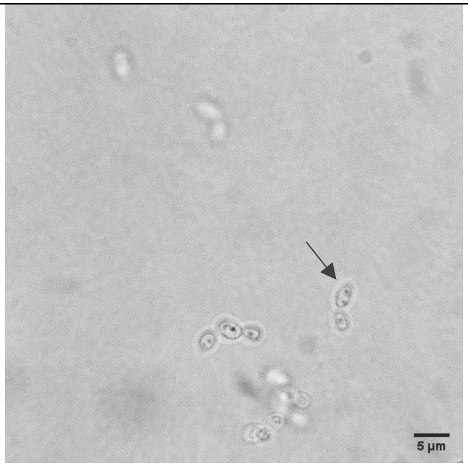	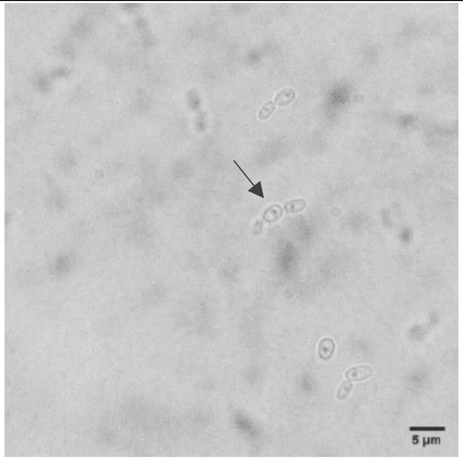	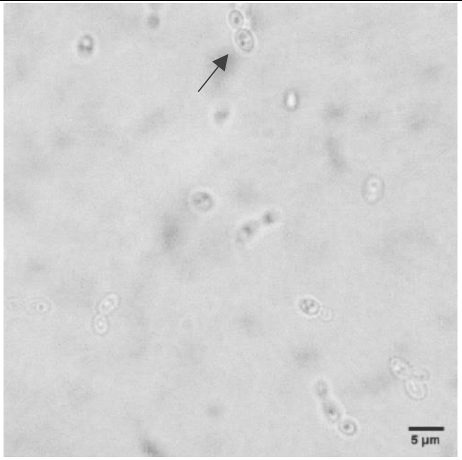	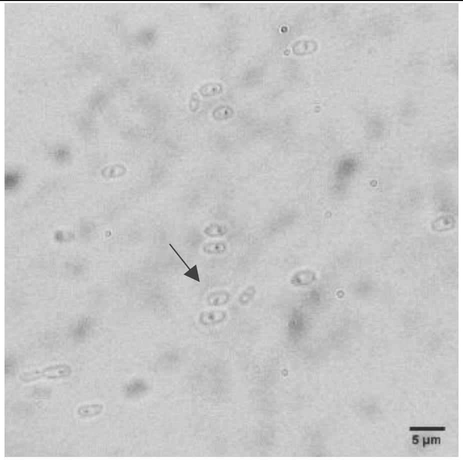
600	no growth/cells	no growth/cells	no growth/cells	no growth/cells

Figure S2. (Continued).

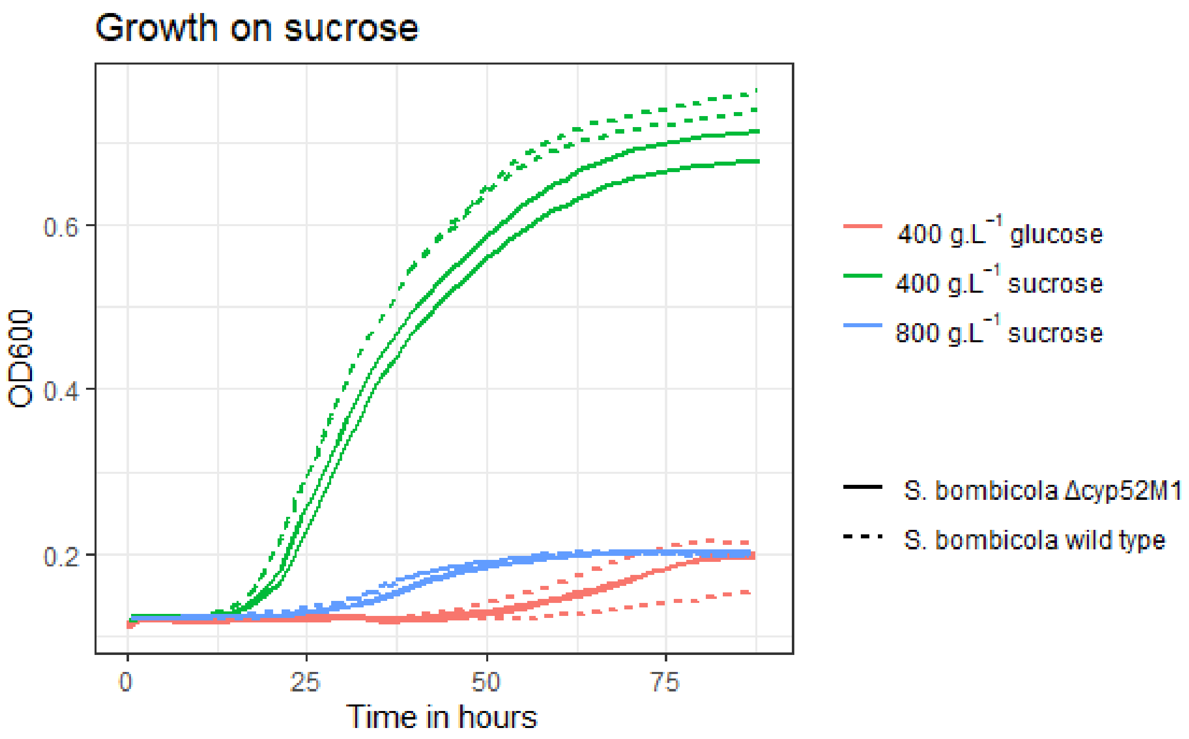

Figure S3. Growth curves (OD_{600}) of a *S. bombicola* wild-type (dotted line) and *Δcyp52M1* strain (full line) on SD CSM medium with 400 g·L^{-1} glucose (red), 400 g·L^{-1} sucrose (green) or 800 g·L^{-1} sucrose (blue) (n=2).

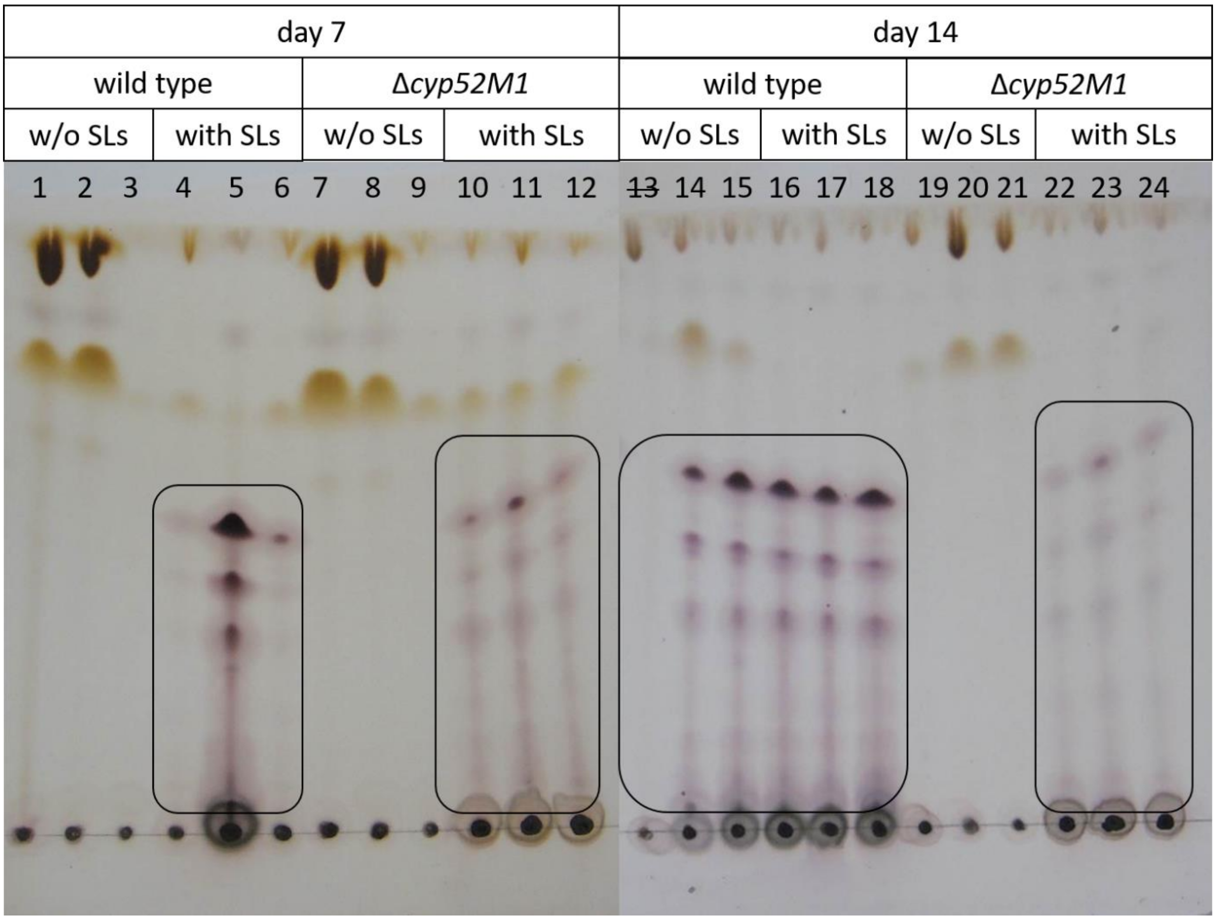

Figure S4. TLC analysis of cell culture broths at day 7 and day 14 of a *S. bombicola* wild-type and a *Δcyp52M1* strain on SD CSM medium with 20 g·L^{-1} rapeseed oil as sole carbon source, with and without the addition of 1 g·L^{-1} sophorolipids (SLs) (n=3). Spots of SLs are purple and framed. Colony 1 of the wild type w/o SLs (lane 13) does not show SL production as this replicate was contaminated after 7 days.

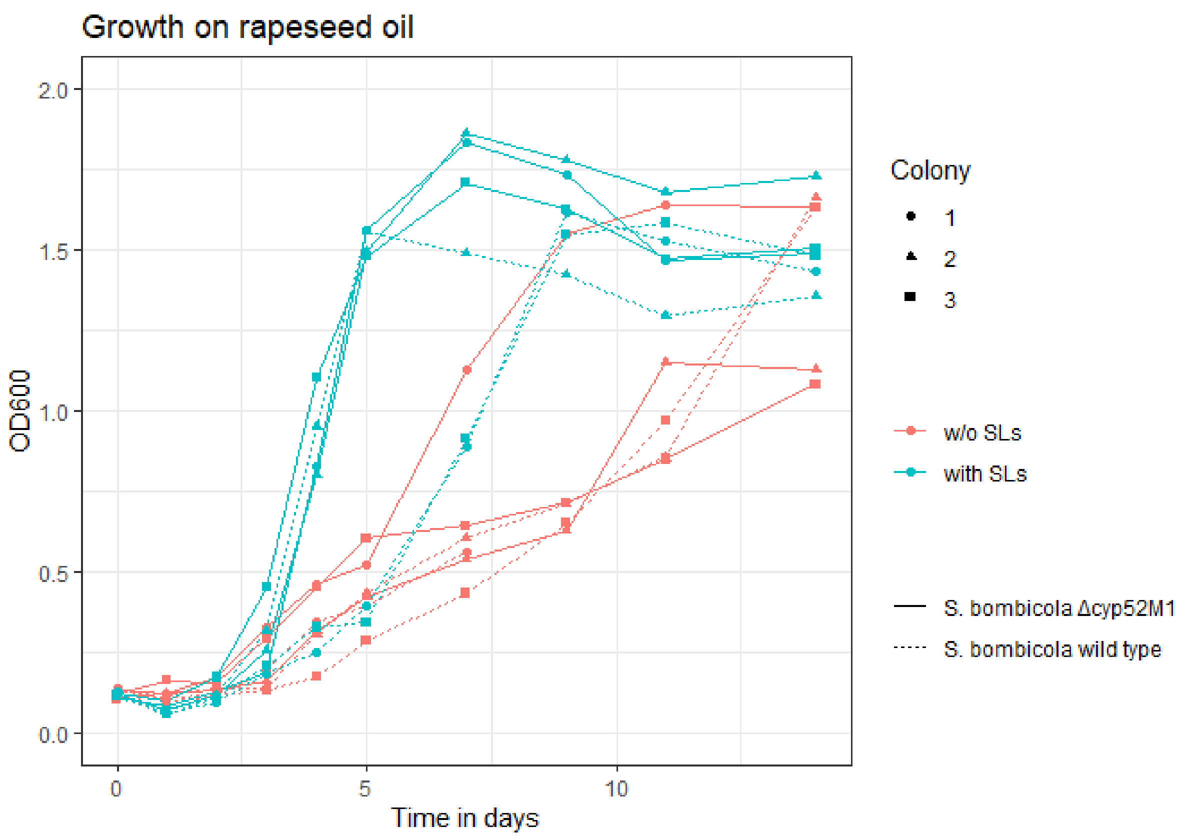

Figure S5. Growth curves (OD_{600}) of a *S. bombicola* wild-type (dotted line) and *Δcyp52M1* strain (full line) on the SD CSM medium with 20 g·L^{-1} rapeseed oil as the sole carbon source, with (blue) and without (red) the addition of 1 g·L^{-1} SLs (14 days, n=3). Datapoints of colony 1 of the wild type on rapeseed oil w/o SLs after day 7 are not shown due to contamination of this replicate.

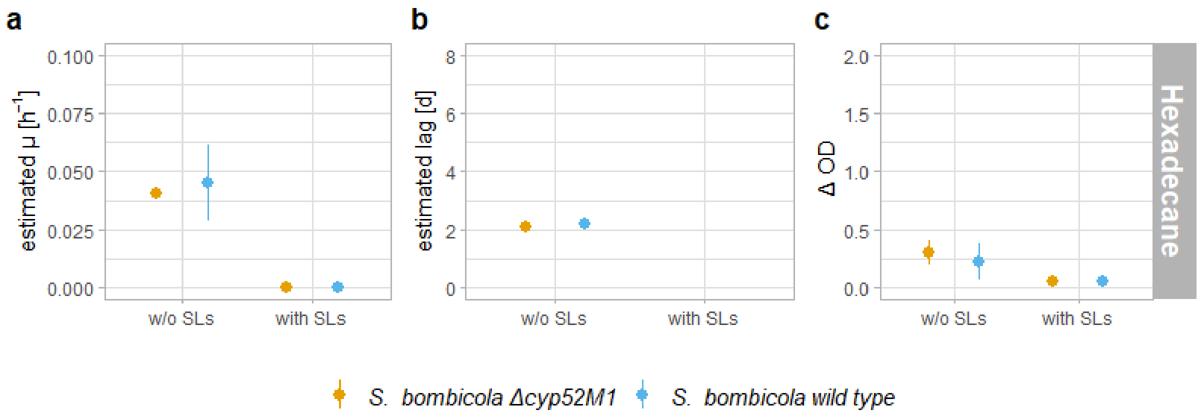

Figure S6. Scatterplots of the estimated growth parameters of a sophorolipid deficient *S. bombicola Δcyp52M1* strain (yellow dots, left) and a sophorolipid producing *S. bombicola* wild-type strain (blue dots, right) on hexadecane (n=3 or 2) in the presence ('with') or absence ('w/o') of 1 g·L^{-1} wild-type sophorolipid mixture. The depicted error bars represent the standard deviation. **(a)** The estimated growth rate μ [h^{-1}], **(b)** the estimated duration of the lag phase [d] (cultures that lagged till the end of the experiment, are not depicted on the plot) and **(c)** the ΔOD (maximum measured OD_{600} value minus value at t_0) at day 7.

Table S1. Influence of pH on the solubility [$g \cdot L^{-1}$] of different forms of SLs. The solubility of purified lactonic and acidic SLs was determined in buffered (KH_2PO_4/K_2HPO_4) aqueous solutions set at pH values of 3, 5, 7, and 8.5 with HCl and NaOH respectively. Minor amounts of the respective SLs were added until the products could not be solubilised anymore and precipitation occurred. When this occurred, the solutions were placed at 30 °C and more SLs were added to saturation if this slight heating again resulted in disappearance of the precipitate. This was performed on an analytical balance in order to determine the amount of product that could be solubilised at a certain pH.

pH	3	5	7	8.5
Acidic non-acetylated SLs solubilised [$g \cdot L^{-1}$]	75	93	187	185
Lactonic di-acetylated SLs solubilised [$g \cdot L^{-1}$]	25	42	69	136

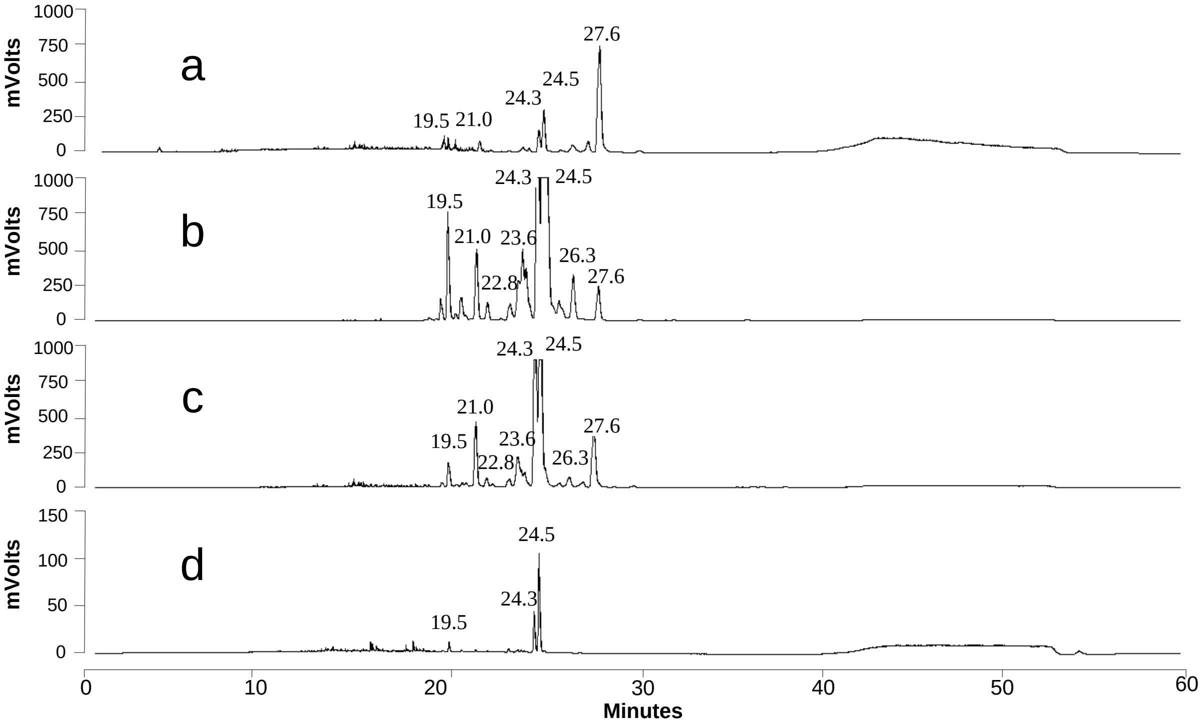

Figure S7. HPLC-ELSD chromatograms of samples from a *S. bombicola* culture on the SL medium with di-acetylated lactonic SLs (27.6 min) as the sole carbon source taken after **(a)** 1 day, **(b)** 8 days, **(c)** 18 days, and **(d)** 30 days of cultivation. Identification of the major peaks detected are listed in the table below.

Table S2. Identification of the major peaks detected in Figure S7 after HPLC-MS analysis. The detected masses (*m/z*) correspond to the $[M-H]^-$ adduct of the identified molecules. The most prominent ones are framed. Several peaks per unique m/z are detected at different retention times, because terminal (ω) and subterminal (ω-1) hydroxylated intermediates correspond to the same MW, but elute differently. The same is true for mono-acetylated derivatives, which can carry the acetyl group on the internal or external glucose moiety.

retention time	m/z	identity		acetylation
19.0	619	acidic SL	C18:2	non
19.5	621	acidic SL	C18:1	non
20.2	623	acidic SL	C18:0	non
21.0	663	acidic SL	C18:1	mono
21.6	663	acidic SL	C18:1	mono
22.8	603/663	lactonic/acidic SL	C18:1	non/mono
23.6	705	acidic SL	C18:1	di
23.7	643 and 703	lactonic/acidic SL	C18:2	mono/di
24.3	645 and 705	lactonic/acidic SL	C18:1	mono/di
24.5	645	lactonic SL	C18:1	mono
26.3	647 and 707	lactonic/acidic SL	C18:0	mono/di
27.0	687	lactonic SL	C18:1	di
27.6	687	lactonic SL	C18:1	di

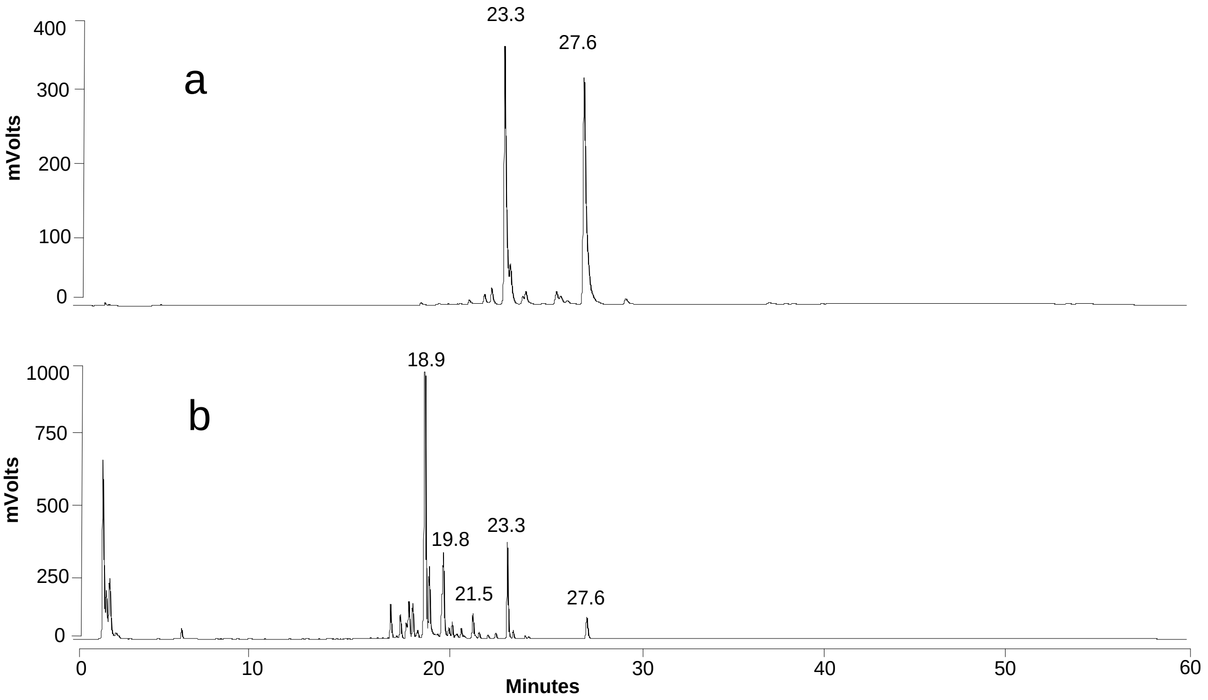

Figure S8. HPLC-ELSD chromatograms of samples from an extracellular activity assay with a *S. bombicola Δsble* strain (unconcentrated secretome) incubated with di-acetylated lactonic SLs (27.6 min) after **(a)** 16 days and **(b)** 32 days of incubation. Because this *Δsble* strain produces (high amounts) of (di-acetylated) acidic SLs (peak at 23.3 min), which are well water soluble, removal of the cells from the culture medium does not lead to removal of these SLs. These were therefore present in the assay from the beginning of the experiment, making it harder to assess if ring opening of lactonic SLs also occurs in the absence of the SBLE enzyme, but ring-opening can be concluded as the ratio between the acidic (23.3 min) and lactonic (27.6 min) SLs clearly increased.

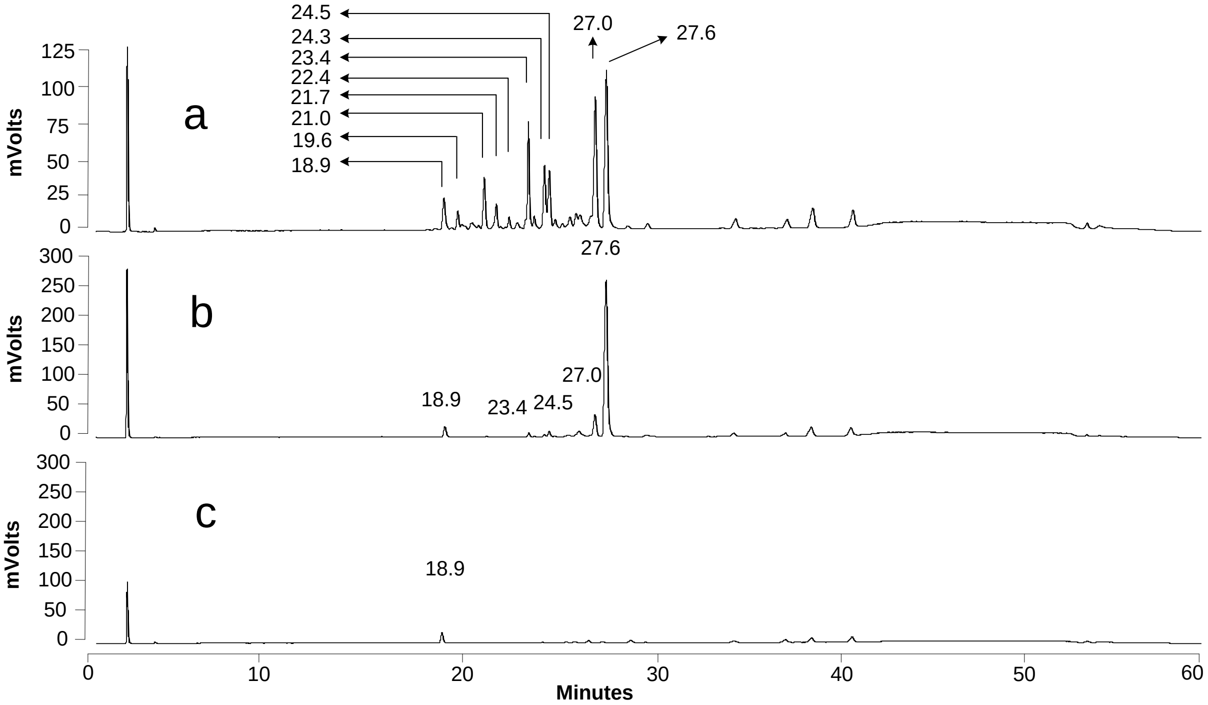

Figure S9. HPLC-ELSD chromatograms of **(a)** an enzyme assay of the wild-type lysate and di-acetylated lactonic SLs (27.6 min) after 6 days of incubation. **(b)** and **(c)** correspond to the controls for SLs and the lysate respectively. The peak at 18.9 minutes corresponds to an internal standard (C12:0-OH). Identification of the major peaks detected are listed in the table below.

Table S3. Identification of the major peaks detected in Figure S9 after HPLC-MS analysis. The detected masses (*m/z*) correspond to the $[M-H]^-$ adduct of the identified molecules. The most prominent ones are framed.

retention time	m/z	identity		acetylation
18.9	216	hydroxylated fatty acid	C12:0	
19.6	621	acidic SL	C18:1	non
20.4	637	acidic SL	C16:0	mono
20.8	623	acidic SL	C18:0	non
21.0	663	acidic SL	C18:1	mono
21.7	663	acidic SL	C18:1	mono
22.4	665	acidic SL	C18:0	mono
23.4	645 and 705	lactonic/acidic SL	C18:1	mono/di
23.8	645	lactonic SL	C18:1	mono
24.3	645 and 705	lactonic/acidic SL	C18:1	mono/di
24.5	645	lactonic SL	C18:1	mono
26.24	647	lactonic SL	C18:0	mono
27.0	687	lactonic SL	C18:1	di
27.6	687	lactonic SL	C18:1	di
38.9	255	fatty acid	C16:0	
41.0	283	fatty acid	C18:0	
